# Human CD56^dim^CD16^dim^ Cells As an Individualized Natural Killer Cell Subset

**DOI:** 10.3389/fimmu.2017.00699

**Published:** 2017-06-19

**Authors:** Mathieu Amand, Gilles Iserentant, Aurélie Poli, Marwan Sleiman, Virginie Fievez, Isaura Pilar Sanchez, Nicolas Sauvageot, Tatiana Michel, Nasséra Aouali, Bassam Janji, Claudia Milena Trujillo-Vargas, Carole Seguin-Devaux, Jacques Zimmer

**Affiliations:** ^1^Department of Infection and Immunity, Luxembourg Institute of Health (LIH), Esch-sur-Alzette, Luxembourg; ^2^Grupo de Inmunodeficiencias Primarias, Facultad de Medicina, Universidad de Antioquia UdeA, Medellín, Colombia; ^3^Grupo de Investigaciones Biomédicas UniRemington, Facultad de Ciencias dela Salud, Corporación Universitaria Remington CUR, Medellín, Colombia; ^4^Luxembourg Competence Centre in Methodology and Statistics, Luxembourg Institute of Health, Luxembourg City, Luxembourg; ^5^Department of Oncology, Luxembourg Institute of Health, Luxembourg City, Luxembourg

**Keywords:** natural killer cells, subsets, CD56^dim^ natural killer cells, human, humanized mouse model

## Abstract

Human natural killer (NK) cells can be subdivided in several subpopulations on the basis of the relative expression of the adhesion molecule CD56 and the activating receptor CD16. Whereas blood CD56^bright^CD16^dim/−^ NK cells are classically viewed as immature precursors and cytokine producers, the larger CD56^dim^CD16^bright^ subset is considered as the most cytotoxic one. In peripheral blood of healthy donors, we noticed the existence of a population of CD56^dim^CD16^dim^ NK cells that was frequently higher in number than the CD56^bright^ subsets and even expanded in occasional control donors but also in transporter associated with antigen processing-deficient patients, two familial hemophagocytic lymphohistiocytosis type II patients, and several common variable immunodeficiency patients. This population was detected but globally reduced in a longitudinal cohort of 18 HIV-1-infected individuals. Phenotypically, the new subset contained a high percentage of relatively immature cells, as reflected by a significantly stronger representation of NKG2A^+^ and CD57^−^ cells compared to their CD56^dim^CD16^bright^ counterparts. The phenotype of the CD56^dim^CD16^dim^ population was differentially affected by HIV-1 infection as compared to the other NK cell subsets and only partly restored to normal by antiretroviral therapy. From the functional point of view, sorted CD56^dim^CD16^dim^ cells degranulated more than CD56^dim^CD16^bright^ cells but less than CD56^dim^CD16^−^ NK cells. The population was also identified in various organs of immunodeficient mice with a human immune system (“humanized” mice) reconstituted from human cord blood stem cells. In conclusion, the CD56^dim^CD16^dim^ NK cell subpopulation displays distinct phenotypic and functional features. It remains to be clarified if these cells are the immediate precursors of the CD56^dim^CD16^bright^ subset or placed somewhere else in the NK cell differentiation and maturation pathway.

## Introduction

Natural killer (NK) cells are the founding members of the innate lymphoid cell family. Their main properties are (i) natural cytotoxicity against tumor cells or infected cells without prior immunization, (ii) antibody-dependent cellular cytotoxicity (ADCC) against antibody-coated target cells, and (iii) cytokine and chemokine production and secretion, which not only are important in innate immunity but also influence the subsequent adaptive immune response (1). Considered for decades as exclusively innate lymphocytes, NK cells have been shown to display memory functions as well (2–4). In addition, they can act as suppressive cells under certain conditions (5).

Whereas a totally specific and selective NK cell marker does not exist, human NK cells are usually described as CD3^−^CD56^+^ large granular lymphocytes. They can be divided into several subpopulations based on the expression of CD56 (an adhesion molecule) and the ADCC-mediating FcγRIIIA receptor CD16 (6–8). In peripheral blood, the numerically major subpopulation is CD56^dim^CD16^bright^ (≥90% of total NK cells) and is frequently described as the most cytotoxic subset, whereas CD56^bright^CD16^dim/−^ NK cells are abundant cytokine producers. Although this is still debated, the majority of authors consider the latter as the immature precursor cells of the CD56^dim^CD16^bright^ population. Peripheral blood furthermore contains some CD56^dim^CD16^−^ and CD56^−^CD16^bright^ NK cells (9–11).

From the functional point of view, NK cells degranulate their cytotoxic vesicles upon encounter with susceptible target cells, a phenomenon that can be measured by flow cytometry with an antibody directed against the vesicle-associated protein CD107a and that reflects cytotoxic activity (12). Furthermore, it has been demonstrated that upon activation, several CD56^dim^CD16^bright^ NK cells lose the expression of CD16 through metalloprotease-mediated shedding and become CD56^dim^CD16^−^ (13), so that the highest percentage of CD107a^+^ degranulating cells is found among the latter population.

The phenotype of the various NK cell subpopulations in terms of repertoires of activating and inhibitory receptors as well as cytokine/chemokine receptors and adhesion molecules is not the same, and this is the basis for their different functional and migratory behavior (14). Thus, for example, CD56^bright^CD16^dim/−^ NK cells are the predominant subset in lymph nodes (LN) (15).

The best studied NK cell inhibitory receptors are specific for human leukocyte antigen (HLA) class I molecules; whereas the killer immunoglobulin receptors (KIR) recognize groups of classical HLA class I alleles, NKG2A binds to the non-polymorphic and non-classical molecule HLA-E. If a developing NK cell expresses one or several self-specific inhibitory receptors, it becomes licensed (educated) and functional (16). In the absence of such a receptor, the NK cell remains unlicensed and hypo-responsive, although it is now recognized that these cells can rapidly become efficient if appropriately stimulated, and that the unlicensed status may actually be advantageous under certain conditions (17).

Natural killer cells go through several maturation stages, progressively losing NKG2A but acquiring KIR and CD57 until reaching the terminal CD56^dim^KIR^+^CD57^+^ phenotype. These cells are functional after interaction with target cells but do not proliferate well any more (18). Although a truly specific maturation marker is still missing, CD226 (DNAM-1) is considered as a molecule characterizing educated, self-specific inhibitory receptor expressing NK cells (18).

By closely examining CD56 versus CD16 flow cytometry dot plots, we noticed the presence of a CD56^dim^CD16^dim^ population numerically minor compared with the CD56^dim^CD16^bright^ NK cells but nevertheless clearly identifiable in a majority of healthy donors (HD). These cells were expanded in transporter associated with antigen processing (TAP)-deficient patients, in some familial hemophagocytic lymphohistiocytosis type II (FHL II), and common variable immunodeficiency (CVID) patients but not in HIV1-infected patients. We also investigated three cases of multiple myeloma (MM) in this regard.

Human TAP deficiency is an autosomal recessive immune defect characterized by a very low cell surface expression of HLA class I molecules and clinically by chronic bacterial infections of the respiratory tract, bronchiectasis, and granulomatous skin lesions, sometimes accompanied by a midface involvement leading to a total destruction of the nasal cartilage (19, 20). Approximately 30 cases have been described to date, but there are probably many more if one considers the high number of idiopathic bronchiectasis cases (21) and the often reduced access to healthcare in regions where consanguinity is frequent. NK cells are numerically normal in this disease but overexpress inhibitory receptors and combinations thereof (22, 23). Functionally, NK cells are hypo-responsive at baseline (they are not educated due to the lack of HLA class I molecules in the environment) but auto-aggressive after cytokine-mediated activation, thus potentially contributing to the chronic inflammatory state of this condition (20, 22, 24).

Familial hemophagocytic lymphohistiocytosis corresponds to a group of rare autosomal recessive immunodeficiencies with defective cytotoxic cells and an over-activation of macrophages. They are the so-called “cytokine storm” syndromes, as the macrophages are activated by very high levels of pro-inflammatory cytokines (25–27). Among the five types described, FHL II is due to a mutation in the perforin gene (25–27).

Common variable immunodeficiency is more frequent and is described as a defect in immunoglobulin production with or without T cell abnormalities (28). A recent study by Ebbo et al. (29) concluded that the clinical severity of the disease is most important in patients with an additional severe NK cell lymphopenia, implicating a protective role of NK cells when present in normal numbers.

Numerous effects of HIV-1 infection on NK cells have been described, and they are only partly restored by combined antiretroviral therapy (cART). Notably, an increase in the CD56^−^CD16^bright^ population is frequently observed, these cells being functionally deficient (30–32).

In this paper, we report the phenotypic and functional characterization of the CD56^dim^CD16^dim^ NK cell subset.

## Materials and Methods

### Sample Collection

Blood samples from HD, HIV-1-infected patients, and TAP-deficient patients were collected, and peripheral blood mononuclear cells (PBMC) were isolated by centrifugation over a Ficoll-Hypaque gradient with Lymphoprep (Elitech). PBMC were either used immediately or frozen (10 × 10^6^ cells/ml) in liquid nitrogen using Recovery Cell Culture Freezing Medium (Invitrogen). Blood samples of HIV-1-infected patients were processed in a BSL3 laboratory. Longitudinal samples of HIV-1-infected patients when viremic before antiretroviral therapy and aviremic under cART for a minimum of 12 months and with a viral load <40 copies/ml were collected (Table S1 in Supplementary Material). Collection was done in accordance with the Declaration of Helsinki from the HD and the patients who each gave informed consent. The study was approved by the National Research Ethics Committee of Luxembourg (CNER, approval numbers 201109/05 and 201209/01). For the pediatric FHL II and CVID patients from Colombia, parents signed the informed consent forms (ethics approval numbers 07-07-111 and 10-7-311, respectively). Only fresh blood was used for this part of the study.

### Multicolor Flow Cytometry

Fresh or thawed cells were stained with conjugated antibodies, listed in Table S2 in Supplementary Material, for 30 min at 4°C in the dark. Stained blood was incubated with BD FACS Lysing Solution (BD Biosciences) for 10 min to lyse red blood cells. After two washes, samples were read on a FACS Fortessa SORP 5 laser instrument (BD Biosciences) and analyzed with the Kaluza Flow Cytometry Analysis Software (Beckman Coulter). Dead cells, monocytes, and T and B lymphocytes were gated out, and NK cell subpopulations were analyzed based on the differential expression of CD56 and CD16. In order to reduce inter-experimental variations, an HD sample was included in each staining in parallel to samples from all patient cohorts.

### Degranulation Assay

Total PBMC or sorted NK cell subsets were incubated for 5 h with K562 cells at an E:T ratio of 7:1. After 1 h of incubation, GolgiStop™ and GolgiPlug™ (BD Biosciences) were added. Negative and positive controls without K562 cells were also included. PMA (*Invivo* Gen) and ionomycin (Life Technologies) were used for the positive condition at 50 and 500 ng/ml, respectively (data not shown). The anti-CD107a antibody was incubated with the cells during the 5 h of incubation. Finally, surface staining and IFN-γ intracellular staining (Table S2 in Supplementary Material) were performed.

For cell sorting, fresh or cryopreserved PBMC from HD were used. NK cells were separated using the MACS NK Cell Isolation Kit (Miltenyi Biotec) according to the manufacturer’s instructions. NK cells were stained with anti-CD56 (clone NCAM16.2) and anti-CD16 (clone VEP13) antibodies. In order to avoid NK cell activation, the anti-CD16 clone VEP13 mAb was used for the cell sorting (33). The CD56^dim^CD16, CD56^dim^CD16^dim^, and CD56^dim^CD16^bright^ subpopulations were aseptically sorted on a FACSAria cell sorter (BD Biosciences) and rested overnight in RPMI-1640 medium supplemented with 10% FBS and antibiotics prior to the degranulation assay. Before the degranulation assays, NK cells were restained with anti-CD56 (clone NCAM16.2) and anti-CD16 (clone VEP13) antibodies.

The myeloid leukemia cell line K562 was purchased from the ECACC and cultured in RPMI-1640 medium supplemented with 10% FBS and antibiotics.

### Generation of NSG and NSG HLA-A2 Humanized Mice

NSG (NOD/LtSz-scid/IL2Rγnull) and NSG HLA-A2 (NOD.Cg-Prkdcscid Il2rgtm1Wjl Tg (HLA-A/H2-D/B2M)1Dvs/SzJ) mice were purchased from Jackson Laboratory, USA. Mice were bred and kept in a specific pathogen-free animal facility. All animal experiments were performed in accordance with the Animal Welfare Committee of LIH (protocol number LRTV 1402) and complied with the national legislation and guidelines for animal experimentation. Humanized NSG and NSG HLA-A2 mice were generated as previously described (34). Six months post-transplantation, mice were euthanized. Tissues and blood samples were processed immediately. LN, spleen, and bone marrow were dissociated with syringes and passed through a nylon cell strainer to obtain single-cell suspensions. Lungs were digested 45 min at 37°C with collagenase A and DNase I recombinant grade I (Sigma-Aldrich) in HBSS (Lonza). Single-cell suspensions were obtained by passing the digested tissue through a 18 G needle and a nylon cell strainer. Red blood cells were lysed using human erythrocyte lysing solution, and samples were washed twice with RPMI-1640. Cells were re-suspended in FACS buffer (PBS, 5% FBS) and stained with the appropriate antibodies as described above.

### Statistics

All results presented in this paper were expressed as mean ± SEM, with the number of biological replicates indicated for each cohort either in the text and/or in the figure legends. A probability level of ≤0.05 was considered significant. We used Wilcoxon matched-pairs signed rank tests for the comparisons between individual NK cell subsets within a cohort and Mann–Whitney *t*-tests for the comparison of individual NK cell subsets between HD and patients. Wilcoxon matched-pairs signed rank tests were also used for the comparison of individual NK cell subsets between longitudinal samples of HIV-1-infected patients when viremic naïve to cART and aviremic under cART. All graphs and statistical analyses were performed using Graphpad Prism 5.0 (Graphpad Software, La Jolla, CA, USA).

## Results

**Figure 1:**
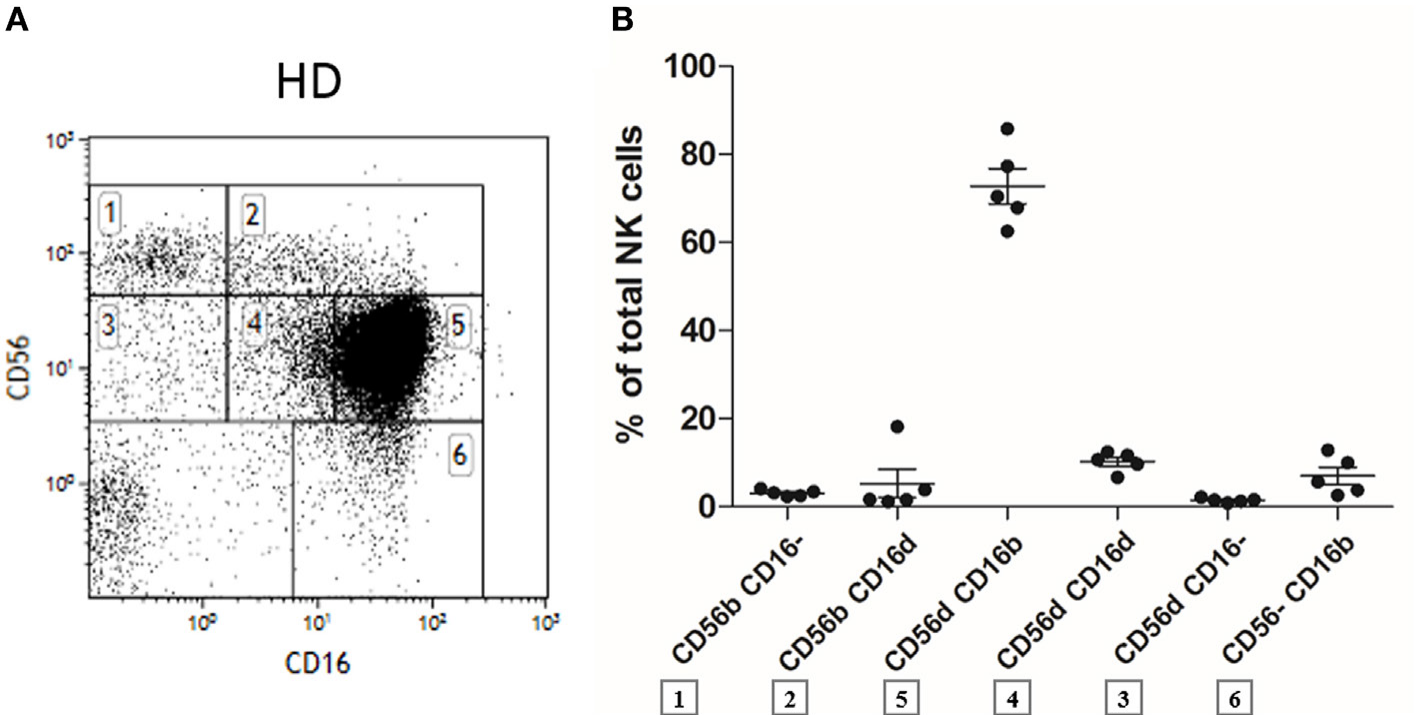
Flow cytometry dot plot of CD56 versus CD16 after gating on alive, single, CD3^−^CD14^−^CD19^−^ blood cells from fresh peripheral blood mononuclear cells (PBMC) of a representative healthy donor **(A)**. Percentages of the different natural killer (NK) cell subsets relative to the total NK cell population (100%) from fresh PBMC of a series of healthy donors (*n* = 5) **(B)**.

On CD16 versus CD56 flow cytometry dot plots of fresh human PBMC (after gating out CD3^+^ T cells, CD19^+^ B cells, and CD14^+^ monocytes), the five usual NK cell subpopulations appeared: CD56^bright^CD16^−^, CD56^bright^CD16^dim^, CD56^dim^CD16^−^, CD56^dim^CD16^bright^, and finally CD56^−^CD16^bright^, as expected and as previously described in the literature (9) (Figure 1A, presented in gates 1, 2, 3, 4 + 5, and 6, respectively). However, by closely looking at such dot plots, we noticed the presence of a numerically minor CD56^dim^CD16^dim^ population in all fresh samples from HD (*n* = 5). To define it, we considered the vertical line starting from the right end of the CD56^dim^CD16^−^ and the left boundary of the CD56^dim^CD16^bright^ cells as shown in Figure 1A (Figure 1A, gate number 4). The percentage of this subset among total NK cells was comparable to or even higher than the ones of the CD56^bright^CD16^−^, CD56^bright^CD16^dim^, CD56^dim^CD16^−^, and CD56^−^CD16^bright^ populations (Figure 1B). All CD56^dim^CD16^dim^ cells were found to express CD7 in each healthy donor and are therefore considered as NK cells and not as CD56^+^CD7^−^ myeloid cells (data not shown) (35).

**Figure 2:**
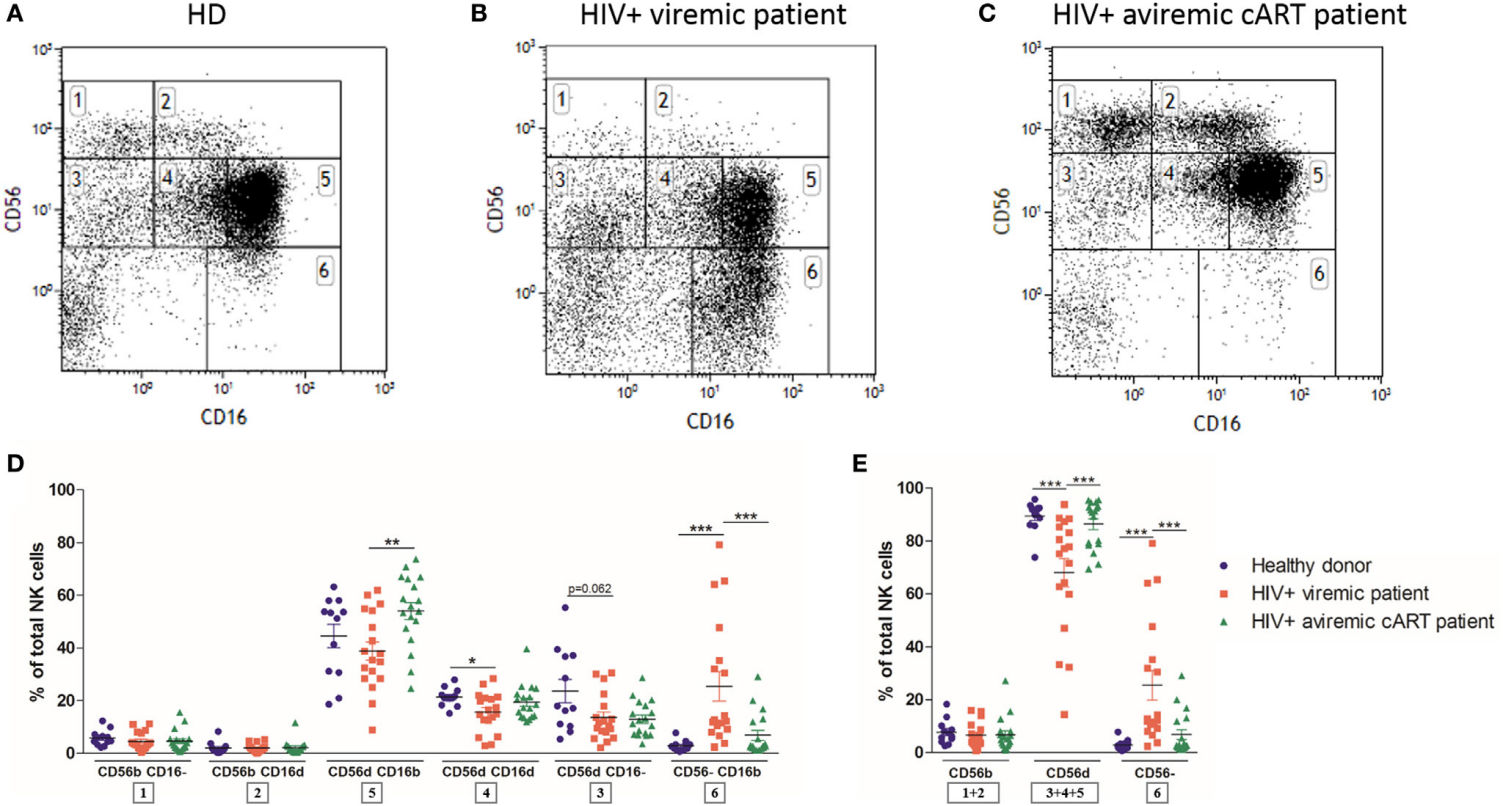
Flow cytometry dot plot of CD56 versus CD16 after gating on alive, single, CD3^−^CD14^−^CD19^−^ blood cells from frozen peripheral blood mononuclear cells (PBMC) of representative healthy donor **(A)**, HIV-1-infected viremic patient **(B)**, and HIV-1-infected aviremic patient under combined antiretroviral therapy (cART) **(C)**. Percentages relative to the total natural killer (NK) cell population (100%) **(D,E)** of different NK cells subsets from frozen PBMC of a series of healthy donors (*n* = 12) and longitudinal paired samples of HIV-1-infected patients when viremic or aviremic under cART (*n* = 18) (*p < 0.05; **p < 0.01; ***p < 0.001).

We next investigated if this NK cell subpopulation could also be detected in immunodeficiencies such as HIV infection and whether it had a distinct phenotype. NK cell populations from frozen PBMC of HD (*n* = 12) and longitudinal paired samples of HIV-1-infected patients when viremic naïve to cART and aviremic under cART (*n* = 18, Figures 2A–C) were analyzed by multicolor flow cytometry. As shown in Figure 2D, the CD56^dim^CD16^dim^ NK cell subset was identified in all HIV-1-infected patients. The appearance of a relatively high percentage of CD56^dim^CD16^−^ and CD56^dim^CD16^dim^ NK cells was observed in thawed PBMC from HD and HIV-infected patients, greater than the one found in fresh samples (36).

As previously described, HIV-1 infection dramatically altered the distribution of NK cell subsets, with, respectively, reducing and increasing the percentages of CD56^dim^ and anergic CD56^−^ NK cells in viremic patients [Figure 2E; (30, 31)]. Both of these abnormalities were reverted by cART (32). Interestingly, among the CD56^dim^ NK cell subsets, only the CD56^dim^CD16^dim^ proportion was significantly reduced by HIV-1 infection and was not restored by cART, suggesting that this subset was more affected by the infection than the other CD56^dim^ subpopulations (Figure 2D).

**Figure 3:**
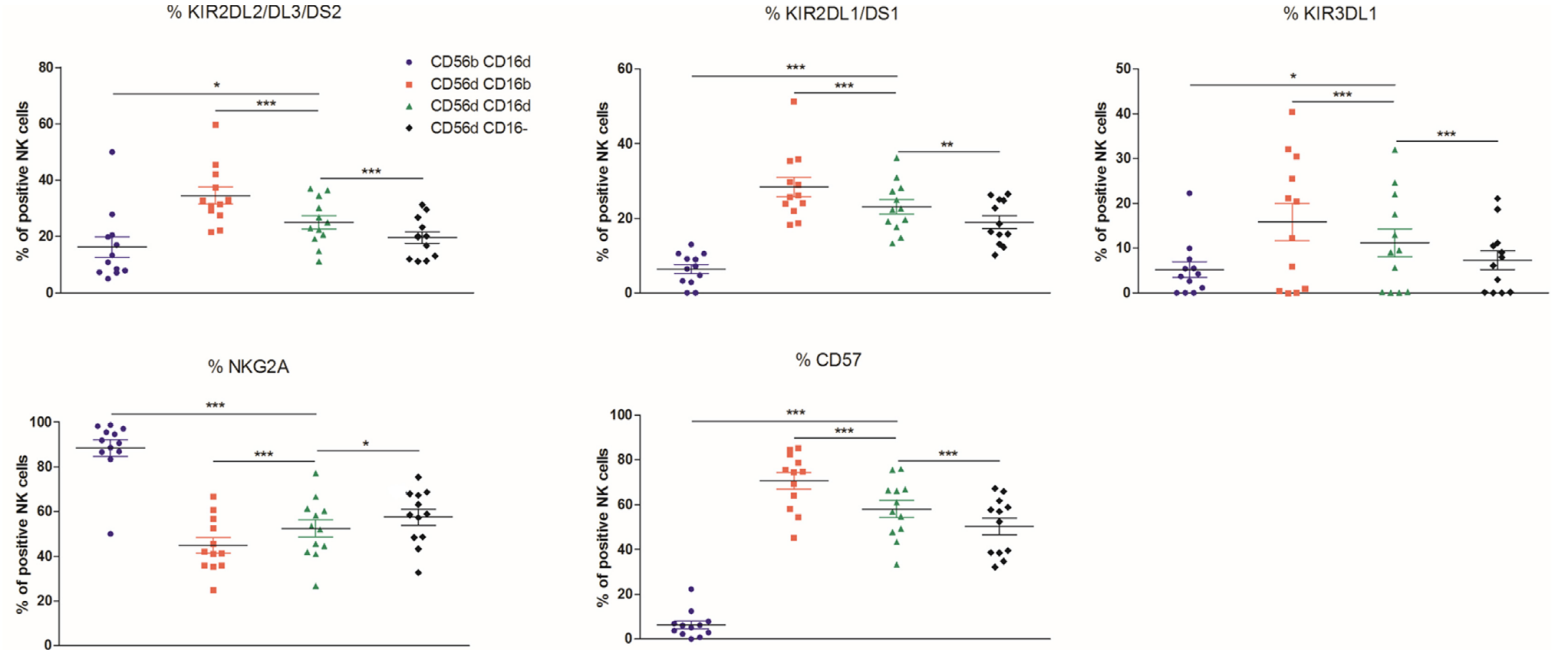
Percentages relative to the total natural killer (NK) cell population (100%) of different blood NK cells subsets expressing the markers KIR2DL2/DL3/DS2, KIR2DL1/DS1, KIR3DL1, NKG2A, and CD57 from frozen peripheral blood mononuclear cells of a cohort of healthy donors (*n* = 12) (*p < 0.05; **p < 0.01; ***p < 0.001).

If one considers that NK cells differentiate from the CD56^bright^ to the CD56^dim^ phenotype *via* the CD56^bright^CD16^dim^ intermediate stage, as shown by Béziat et al., one might expect that the CD56^dim^CD16^dim^ population corresponds to the immediate precursors of the CD56^dim^CD16^bright^ cells (37). On the other hand, CD56^dim^CD16^dim^ cells could also represent an intermediate stage between CD56^dim^CD16^bright^ and CD56^dim^CD16^−^ NK cell subsets. In the HD cohort (*n* = 12), KIR2DL1/DS1, KIR2DL2/DL3/DS2 and KIR3DL1, CD57, NKG2D, SIGLEC-7, CD38, CD244, CD62L, CD8, and CD226 were more expressed on CD56^dim^CD16^bright^ than on CD56^dim^CD16^−^ cells, whereas NKG2A, CD27, CD69, and HLA-DR varied in an opposite manner (Figure 3; Figures S1 and S2 in Supplementary Material), suggesting overall a more mature phenotype of CD56^dim^CD16^bright^ than CD56^dim^CD16^−^ NK cells. We observed systematically an intermediate or equal expression of those markers in CD56^dim^CD16^dim^ NK cells as compared to the former subsets, emphasizing an intermediate phenotype between the CD56^dim^CD16^bright^ and CD56^dim^CD16^−^ populations. In addition, CD56^bright^CD16^dim^ NK cells demonstrated a more immature phenotype than CD56^dim^CD16^dim^ NK cells with a lower expression of KIR2DL1/DS1, KIR2DL2/DL3/DS2, KIR3DL1, CD57 (Figure 3), KLRG1 (Figure S1 in Supplementary Material) and a higher expression of NKG2A (Figure 3), CD27, and CD62L (Figure S1 in Supplementary Material). All the multicolor flow cytometry data are presented in Table S3 in Supplementary Material.

Altogether, this pattern indicates that CD56^dim^CD16^dim^ NK cells may be an intermediate stage between CD56^dim^CD16^bright^ and CD56^dim^CD16^−^ NK cells or between CD56^dim^CD16^bright^ and CD56^bright^CD16^dim^ NK cells.

Although, as previously stated, the use of frozen PBMC can induce the appearance of a higher percentage of CD56^dim^CD16^−^ and CD56^dim^CD16^dim^ NK cells (38), the staining for NKG2A did not vary before and after freezing/thawing in any of the subpopulations analyzed. In the case of CD226 and KLRG1, however, there was a trend toward a higher expression on thawed cells, but only in the CD56^dim^CD16^−^ subset (Figure S3 in Supplementary Material).

**Figure 4:**
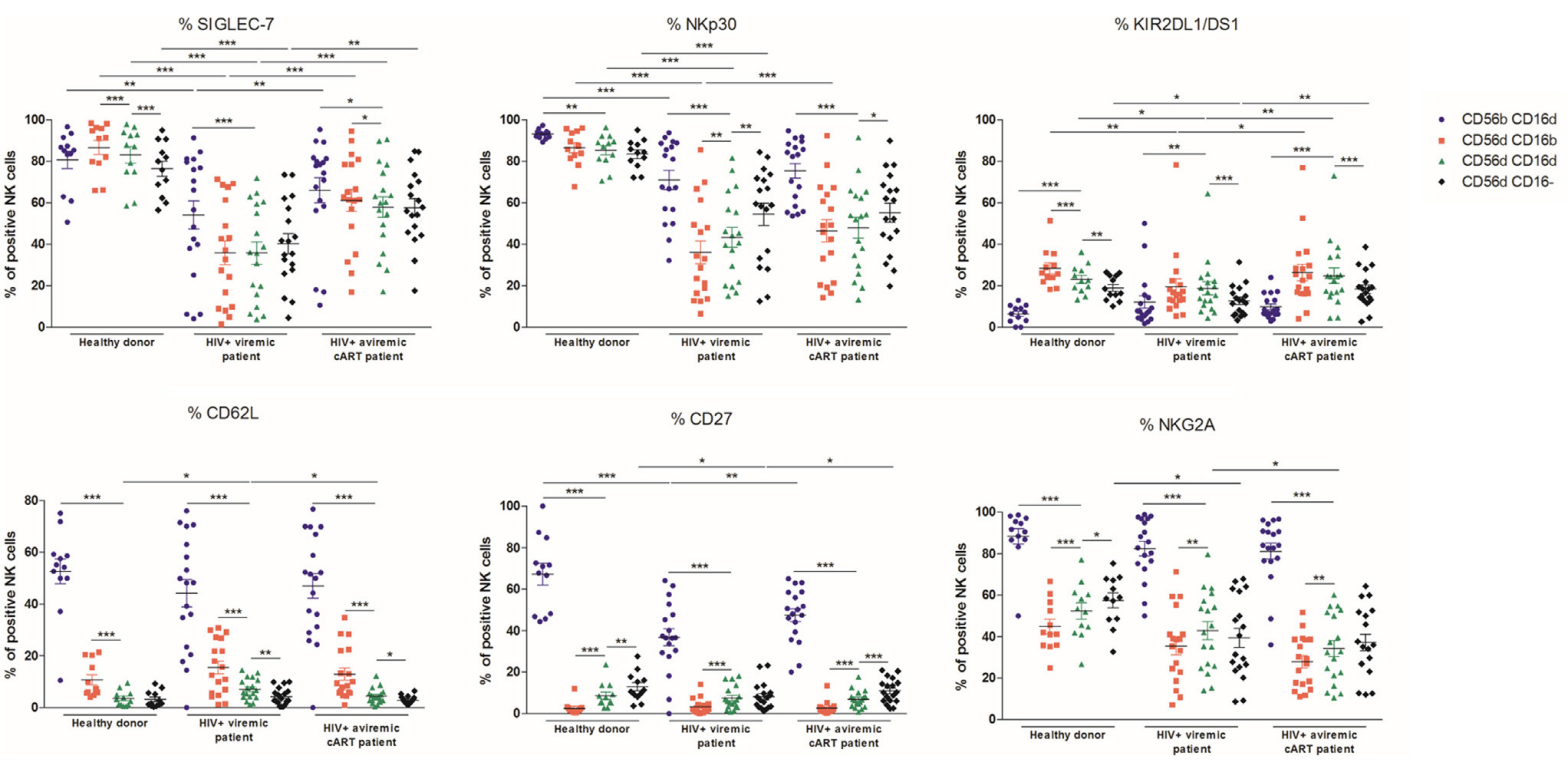
Percentages relative to the total natural killer (NK) cell population (100%) of different blood NK cells subsets expressing the markers SIGLEC-7, NKp30, KIR2DL1/DS1, CD62L, CD27, and NKG2A from frozen peripheral blood mononuclear cells of a series of healthy donors (*n* = 12) and longitudinal paired samples of HIV-1-infected patients when viremic or aviremic under combined antiretroviral therapy (*n* = 18) (*p < 0.05; **p < 0.01; ***p < 0.001).

Since the CD56^dim^CD16^dim^ subset was, among all CD56^dim^ cells, the most affected by HIV-1 infection, we next investigated whether the expression of a large set of markers in this population could be differentially affected and distinguish its phenotype as compared to the other NK cell subsets (Figure 4).

SIGLEC-7, a marker of NK cell functionality, and the activating receptor NKp30 were decreased in viremic patients in CD56^bright^CD16^dim^, CD56^dim^CD16^bright^, CD56^dim^CD16^dim^, and CD56^dim^CD16^−^ NK cell subsets and as previously reported (30, 39, 40). Although the CD56^bright^CD16^dim^ population was slightly affected by HIV-1 infection, the CD56^dim^ subsets, and particularly CD56^dim^CD16^bright^ NK cells, showed a massively decreased expression of SIGLEC-7 and NKp30 in viremic patients. In contrast to HD, in viremic patients, no differences were observed between CD56^dim^ NK subsets in terms of SIGLEC-7 expression, whereas NKp30 was more expressed in the CD56^dim^CD16^−^ subpopulation. Once more, CD56^dim^CD16^dim^ NK cells were systematically intermediate between CD56^dim^CD16^bright^ and CD56^dim^CD16^−^ NK cells (Figure 4; Figures S4 and S5 in Supplementary Material). SIGLEC-7 expression was significantly increased in all the NK subsets in aviremic patients under cART, whereas NKp30 was only significantly restored in CD56^dim^CD16^bright^ NK cells (Figure 4).

In HD, KIR2DL1/DS1 expression was higher in the CD56^dim^ subsets as compared to the CD56^bright^CD16^dim^ NK cells. In the CD56^dim^ subsets, its expression decreased successively between CD56^dim^CD16^bright^, CD56^dim^CD16^dim^, and CD56^dim^CD16^−^ NK cells. HIV-1 infection impaired KIR2DL1/DS1 expression in all the CD56^dim^ subtypes. Furthermore, their expression did not differ anymore between the CD56^dim^CD16^bright^ and CD56^dim^CD16^dim^ subsets in the patients when viremic or aviremic (Figure 4; Figure S6 in Supplementary Material). KIR2DL1/DS1 expression was slightly restored in all CD56^dim^ subsets in patients when aviremic under cART (Figure 4).

CD27, CD62L, and NKG2A were found systematically less expressed in the CD56^dim^ subsets as compared to the CD56^bright^CD16^dim^ NK cells in HD and HIV-1-infected patients. Among the CD56^dim^ subsets, the CD56^dim^CD16^dim^ population significantly increased CD62L expression in patients when viremic and partially decreased it when aviremic under cART. CD27 was found decreased in CD56^bright^CD16^dim^ and CD56^dim^CD16^−^ but not in CD56^dim^CD16^dim^ subtypes during infection. cART restored partially the HD phenotype. NKG2A expression significantly decreased in the CD56^dim^CD16^−^ subset in the viremic patients but was not restored by cART. In contrast to HD, in viremic patients, no differences were observed between CD56^dim^CD16^dim^ and CD56^dim^CD16^−^ NK subsets (Figure 4).

Ultimately, these results indicate that CD56^dim^CD16^dim^ NK cells are, at times, differentially regulated upon HIV-1 infection compared with CD56^dim^CD16^bright^ and CD56^dim^CD16^−^ subsets, therefore reinforcing the hypothesis that they compose a different NK cell subset.

**Figure 5:**
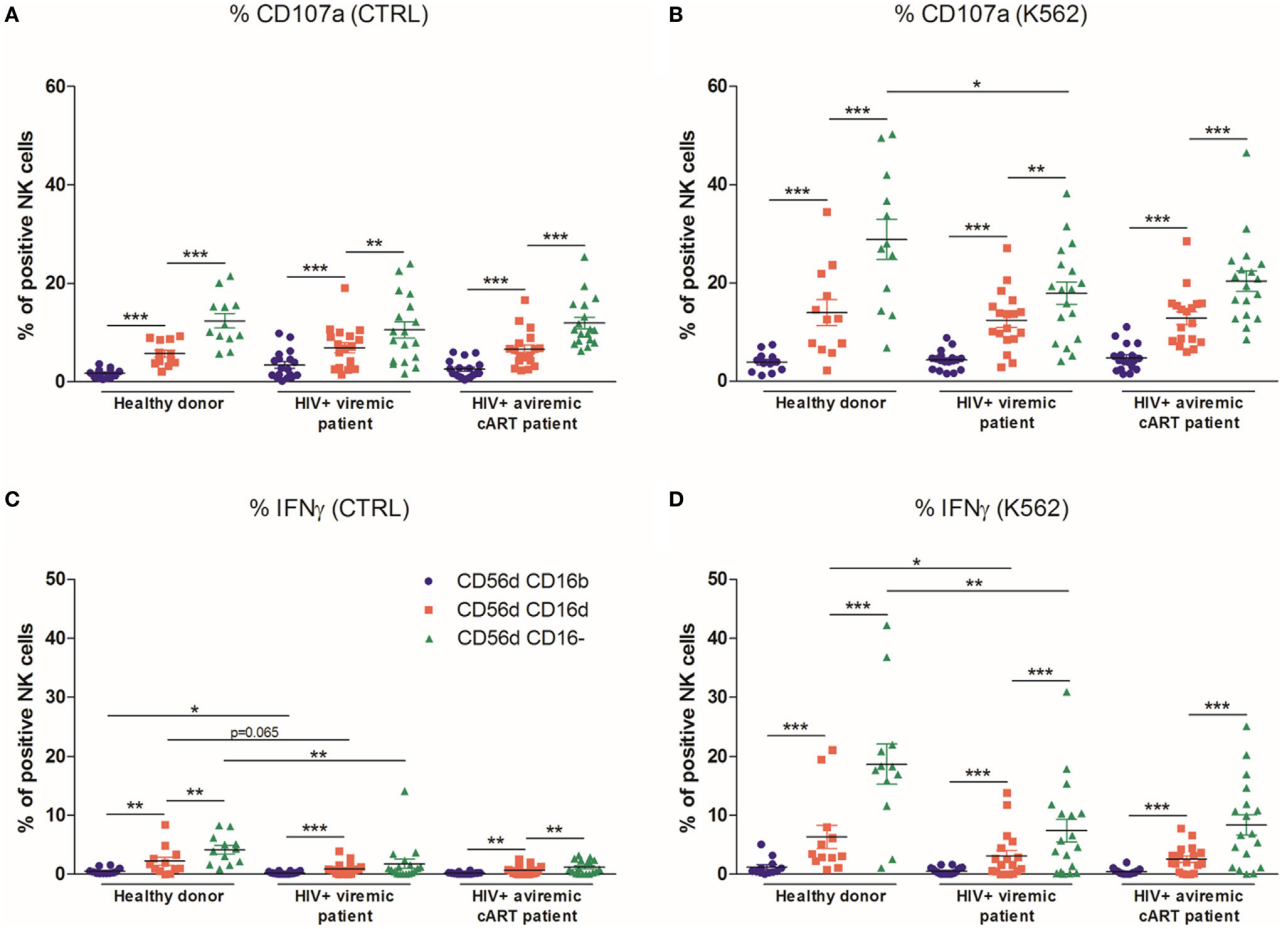
Percentages relative to the total natural killer (NK) cell population (100%) of different blood NK cells subsets expressing CD107a from frozen peripheral blood mononuclear cells (PBMC) of a series of healthy donors (HD) (*n* = 12) and longitudinal paired samples of HIV-1-infected patients when viremic or aviremic under combined antiretroviral therapy (cART) (*n* = 18) at basal levels **(A)** or following stimulation with K562 cells **(B)**. Percentages relative to the total NK cell population (100%) of different blood NK cells subsets producing IFNγ from frozen PBMC of a series of HD (*n* = 12) and longitudinal paired samples of HIV-1-infected patients when viremic or aviremic under cART (*n* = 18) at basal levels **(C)** or following stimulation with K562 cells **(D)** (*p < 0.05; **p < 0.01; ***p < 0.001).

The different subsets were tested for their ability to degranulate and produce IFN-γ against K562 target cells using frozen PBMC. In HD and HIV-1-infected patients, the most proficient degranulating subset were the CD56^dim^CD16^−^ cells as previously described (36), followed by the CD56^dim^CD16^dim^ population, whereas the remaining CD56^dim^CD16^bright^ NK cells did not efficiently degranulate (Figures 5A,B). The CD56^dim^CD16^−^ population was similarly the most effective subset to produce IFN-γ. In viremic HIV-1-infected patients, CD107a expression was found to be impaired in CD56^dim^CD16^−^ NK cells upon stimulation with K562 cells. The IFN-γ production was also impaired in all CD56^dim^ subsets at basal level and in CD56^dim^CD16^dim^ and CD56^dim^CD16^−^ NK cells after stimulation with K562. Combined ART restored neither CD107a expression nor IFN-γ production (Figures 5C,D).

**Figure 6:**
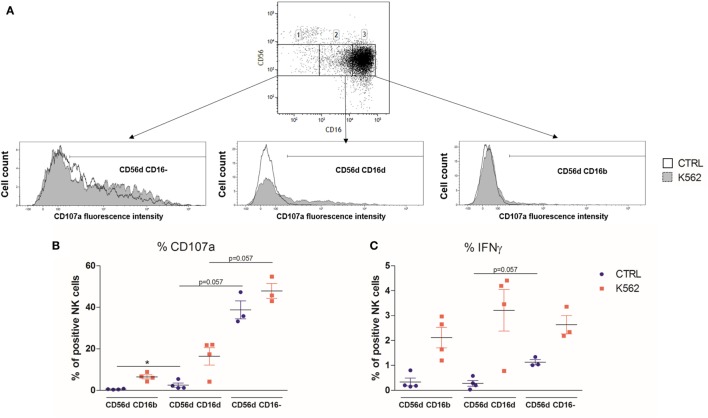
**(A)** Flow cytometry dot plot CD56 versus CD16 after gating on alive, single, CD3^−^CD14^−^CD19^−^ blood cells from fresh peripheral blood mononuclear cells (PBMC) of a representative healthy donor prior to cell sorting **(A)**. The gates 1, 2, and 3 represent the sorted CD56^dim^CD16^−^, CD56^dim^CD16^dim^, and CD56^dim^CD16^bright^ subsets, respectively. Each subset was stimulated with K562 cells, and their degranulation and IFNγ production were assessed by flow cytometry. **(B,C)** Percentages of blood natural killer cells from sorted CD56^dim^CD16^bright^, CD56^dim^CD16^dim^, and CD56^dim^CD16^−^ subtypes expressing CD107a **(B)** and IFNγ **(C)** from fresh PBMC of a series of healthy donors (*n* = 4) at basal levels or following stimulation with K562 cells (*p < 0.05).

Stimulation with K562 can induce the loss of CD16 expression on a large part of NK cells (13, 36). Hence, this type of experiment does not allow to formally discriminate if the CD56^dim^CD16^dim^ NK cells are a new subset or just CD56^dim^CD16^bright^ cells that have begun to lose CD16 expression. To further confirm our results, we sorted the different CD56^dim^ populations separately by starting from fresh HD PBMC (*n* = 4) and then assessed their degranulation and cytokine production in response to K562 cell stimulation (Figure 6A). In one HD, the CD56^dim^CD16^−^ NK cells were almost absent, and no data were obtained for this subset. In line with our previous observations, the CD107a staining showed a tendency toward an increased degranulation from CD56^dim^CD16^bright^ to CD56^dim^CD16^dim^ and CD56^dim^CD16^−^ NK cells. Interestingly, in this experiment, CD56^dim^CD16^−^ NK cells were already able to degranulate without K562 cells at quite a high level (Figure 6B). In terms of IFN-γ production, although we observed the same tendency at basal levels, in the stimulated condition, we were not able to observe significant differences between the three subsets (Figure 6C).

**Figure 7:**
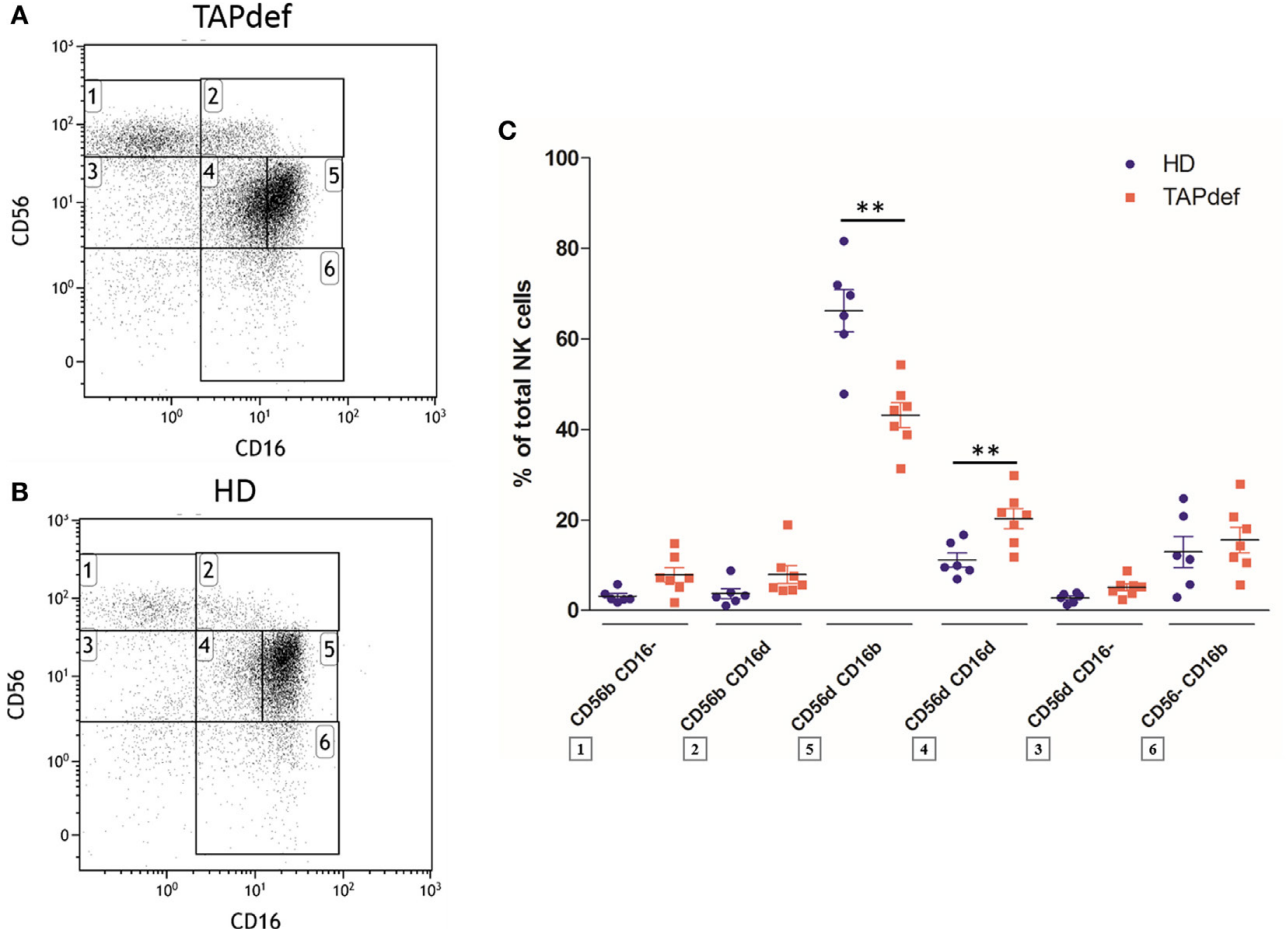
Flow cytometry dot plot of CD56 versus CD16 after gating on alive, single, CD3^−^CD14^−^CD19^−^ blood cells from a representative TAP-deficient patient **(A)** and a representative healthy donor **(B)**. Percentages of the different natural killer (NK) cell subsets relative to the total NK cell population (100%) in a cohort of TAP-deficient patients (*n* = 7) compared with healthy donors (*n* = 6) **(C)** (*p < 0.05; **p < 0.01).

We next investigated the presence of the CD56^dim^CD16^dim^ NK cells in other immunodeficiencies than HIV infection. We found CD56^dim^CD16^dim^ NK cells to be not only present but even expanded in the blood of seven patients with TAP deficiency (Figures 7A,C) compared with normal donors (Figures 7B,C). In parallel, we observed a diminution in the percentage of cells in the CD56^dim^CD16^bright^ population in patients (Figure 7C). This increase in CD56^dim^CD16^dim^ NK cells was also found in two (Figures S7A,B in Supplementary Material) out of four (Figures S7C,D in Supplementary Material) patients with FHL II (Figure S7E in Supplementary Material). In a CVID cohort of 19 patients compared to 14 HD, no significant difference was present in the CD56^dim^CD16^dim^ subset, whereas the mean percentage of CD56^dim^CD16^bright^ NK cells was significantly lower in these patients (Figure S8 in Supplementary Material). To check if the new subset would also be present in a neoplastic disease, we investigated the peripheral blood of three MM patients. Here, no marked CD56^dim^CD16^dim^ subset was present (data not shown).

**Figure 8:**
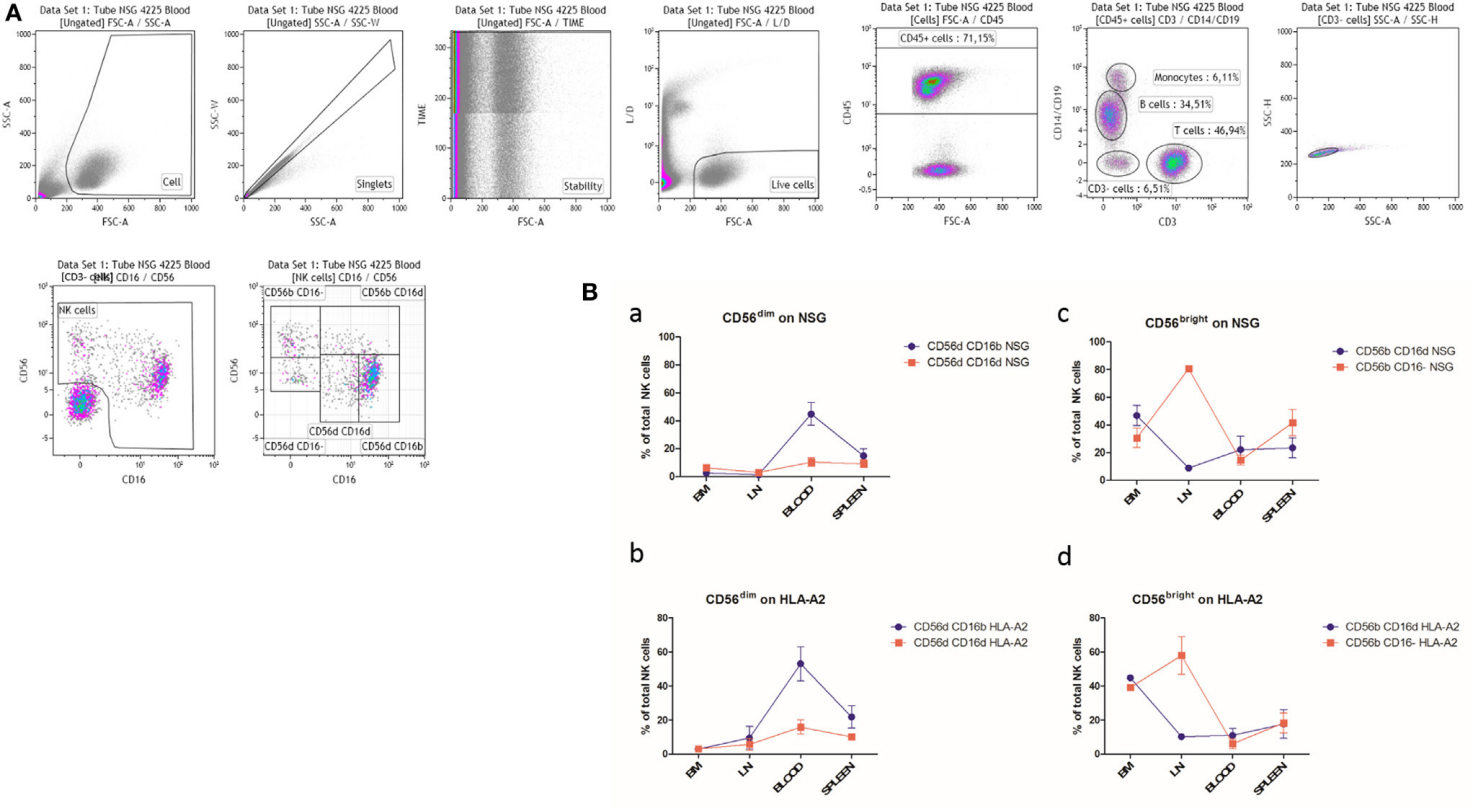
**(A)** Peripheral blood cells from a representative humanized NSG mouse were stained with antibodies as described in Material and Methods. The gate was set on alive single cells positive for human CD45 to exclude contaminating mouse cells. The CD3 versus CD14/CD19 dot plot allows the identification and discrimination of human T cells, B cells, and monocytes. The remaining triple-negative cells were analyzed in a CD56 versus CD16 dot plot to identify the natural killer (NK) cells. CD56^dim^CD16^dim^ NK cells are clearly present. **(B)** Percentages of CD56^dim^ (a, b) and CD56^bright^ (c, d) NK cell subsets in various organs of humanized NSG (NSG; a, c; *n* = 3) and NSG/human leukocyte antigen (HLA)-A2 (HLA-A2; b, d; *n* = 2) mice. BM: bone marrow; LN: lymph nodes.

We next sought to investigate the existence of CD56^dim^CD16^dim^ NK cells in a humanized mouse model, in which immunodeficient NSG mice receive human CD34^+^ hematopoietic stem cells from cord blood after myelosuppression and reconstitute a human immune system within several months (41, 42). Six months after transplantation, three standard NSG mice and two NSG mice transgenic for the classical HLA class I molecule HLA-A2 showed a good engraftment (89–94% of human CD45^+^ cells in spleen) and displayed human monocytes, B cells, T cells, and CD3^−^ cells in peripheral blood (Figure 8A) as previously described (43). NK cell subpopulations defined by CD56 and CD16 expression, with the exception of CD56^−^CD16^bright^ cells in some animals, could be found in peripheral blood, including the newly described CD56^dim^CD16^dim^ subset. A comparable distribution, although with different relative percentages per subpopulation, was observed in spleen (Figure 8B) and lung (data not shown), whereas BM (more or less equal distribution of CD16^−^ and CD16^dim^ cells) and lymph node (large predominance of CD16^−^ cells) NK cells were almost exclusively CD56^bright^ (Figure 8B), similar to what is observed, in the latter case, for original human LN (15).

## Discussion

In this paper, we describe CD56^dim^CD16^dim^ NK cells as a subset of human peripheral blood NK cells and compare it to the other CD56^dim^ populations in terms of phenotype and functions. We show that this subpopulation is present in almost all HD, expanded in TAP deficiency but slightly reduced in HIV-1 infection. The phenotypic characterization based on classical NK cell markers revealed an intermediate position between CD56^dim^CD16^bright^ NK cells and both the CD56^dim^CD16^−^ and the CD56^bright^CD16^dim^ subsets. Indeed, CD56^dim^CD16^dim^ NK cells are clearly less mature than CD56^dim^CD16^bright^ NK cells (as illustrated in particular by the higher expression of NKG2A and the lower levels of CD57, CD226 and KIR), but more mature than the other two populations based on the same arguments just the other way around. Several additional markers are completing this picture. From the functional point of view, CD56^dim^CD16^dim^ cells degranulate, in the presence of K562 target cells, to a higher level than CD56^dim^CD16^bright^ cells but much less than CD56^dim^CD16^−^ cells.

The latter observation was made with thawed PBMC as well as with freshly FACS-sorted and purified NK cells, which validates the procedures using frozen cells. When we phenotyped fresh compared to thawed PBMC, the percentages of the CD56^dim^CD16^−^ and CD56^dim^CD16^dim^ populations slightly increased among the latter, but the phenotype of the various subsets remained the same, except for a trend toward higher expression of CD226 and KLRG1 solely among the thawed CD56^dim^CD16^−^ cells. We conclude that the phenotypic and functional differences found between CD56^dim^CD16^dim^ and CD56^dim^CD16^bright^ NK cells are valid, regardless if fresh or thawed cells were used.

A major problem when studying CD16 is that this molecule is quickly shed from the NK cell surface upon activation due to the action of metalloproteases (13). This could very well explain why the majority of degranulating cells are found within the CD56^dim^CD16^−^ subset. Such a phenomenon has been previously described (36). Furthermore, under these conditions, CD56^dim^CD16^dim^ NK cells are likely a mixture of relatively immature cells and previously CD56^dim^CD16^bright^ cells that are on their way to lose CD16 expression due to activation-induced shedding.

Currently, a linear NK cell differentiation from immature CD56^bright^CD16^−^ through CD56^bright^CD16^dim^ to the mature CD56^dim^CD16^bright^ subsets is admitted by most authors, as most recently discussed by Mace (44), the latter population being further subdivided into more or less terminally differentiated subtypes (9). Based on this model and on their relatively immature phenotype revealed in this study, one might consider the CD56^dim^CD16^dim^ NK cells as the immediate precursors of the CD56^dim^CD16^bright^ cells.

However, recent lineage tracing data are questioning the linear sequence and suggest on the contrary that the CD56^bright^ and CD56^dim^ NK subsets might originate from two different precursors (45, 46). The question is therefore still not definitely resolved and deserves further study. Should this possibility be demonstrated in the future, the new subset could still represent immature precursors of the CD56^dim^CD16^bright^ population.

An interesting possibility to also consider could be that the CD56^dim^CD16^dim^ cells are at the crossroad of all the precursors of the mature CD56^dim^CD16^bright^ subset. This means that they would be composed of (i) descendants of immature CD56^bright^CD16^dim^ cells originating from the CD56^bright^ precursor, (ii) descendants of immature CD56^dim^CD16^−^ cells originating from the CD56^dim^ precursor, and (iii) activated, formerly CD16^bright^ NK cells. This configuration would fit with the fact that both CD56^bright^CD16^dim^ and CD56^dim^CD16^−^ NK cells have a more immature phenotype than CD56^dim^CD16^dim^ cells. Our data so far do not allow to confirm or reject this hypothesis. Similarly, it is not clear if the CD56^dim^CD16^−^ cells are only immature precursors or contain also a fraction of activated mature formerly CD56^dim^CD16^bright^ cells even at baseline. Upon activation with K562 (for example), this population would then be considerably enriched with mature cytotoxic NK cells having lost CD16.

Another point to be discussed is the fact that if one assumes that in the functional assays, the cells with the most immature phenotype were also those that contained the highest fraction of degranulating cells (the CD56^dim^CD16^−^ fraction), this would suggest that immaturity does not necessarily correlate with absence of functionality and would be reminiscent of the high cytokine producing capacity of the phenotypically immature CD56^bright^CD16^−^ NK cells (11). However, to be sure of this, one would have to compare the phenotype of the same donor CD56^dim^CD16^−^ NK cells before and after activation with K562, in order to check if the percentages of NKG2A^+^, KIR^+^, and CD57^+^ cells would vary in this population.

To our knowledge, this CD56^dim^CD16^dim^ population has not been formally described before. Stabile et al. (47) discuss CD56^low^CD16^low^ cells, but a close look at their gating strategy shows that they are actually studying CD56^dim^CD16^−^ NK cells. The CD56^dim^CD16^dim^ population clearly appears in CD16 versus CD56 flow cytometry dot plots in several papers, but it is always gated together with the CD56^dim^CD16^bright^ subset. Likely closely related to this population are the NK cells described by Lima et al. (48), but these authors discriminate their subset on the basis of the expression level of CD56, which is higher than that on the CD56^dim^CD16^bright^ cells. Furthermore, Krzywinska et al. distinguish NK cell subpopulations according to the CD45 isoforms expressed. They also show CD56^dim^CD16^dim^ cells, but here again, the CD56^dim^CD16^−^ subset is included in the gating (49).

Humanized mice are becoming increasingly important in immunology, for example as models allowing HIV infection and subsequent drug evaluation in small animals. The engraftment of NK cells under these conditions is not always optimal, so that some groups use exogenous human IL15/IL15Rα complexes or human cytokine gene knock-ins to improve the NK cell yield (41, 42, 50). Without any of such methods, we reproducibly observed between 6 and 11% of total living human CD45^+^ cells being negative for lineage markers such as CD3, CD14, and CD19. Among them, the CD56^dim^CD16^dim^ population interestingly showed up in all the organs tested and was even sometimes relatively abundant, which might suggest that the immune system of these mice was not yet fully mature at the time of sacrifice, with a relatively high amount of CD56^dim^ NK cells still in the precursor phase. Alternatively, NK cells could be spontaneously activated in this model to some extent and the CD56^dim^CD16^dim^ subset represents cells in the process of completely losing CD16. The NK cells from the humanized mice were quite functional in terms of degranulation but did not produce IFN-γ, as previously described for the NSG model by Rongvaux et al. (42). In addition, it is well known that not all NK cells are cytotoxic and cytokine producers at the same time (51). The various subpopulations could not be FACS-sorted due to the rarity of NK cells in this model that needs further improvement.

The question arises as to why the CD56^dim^CD16^dim^ subset was so dramatically upregulated in TAP deficiency as well as in some cases of FHL II and CVID, and occasionally, in HD. In the former, we have previously described an expansion of the CD56^bright^ population that might be due to a higher amount of precursor cells due to the chronic infectious state of these patients needing a continuous replenishment with new NK cells (52). Under these conditions, it would not be unexpected to also observe an increase in the CD56^dim^CD16^dim^ precursors, and the same could be true for the other primary immunodeficiencies. On the other hand, considering the interesting possibility that CD56^bright^ and CD56^dim^ NK cells originate from two different lineages, the CD56^dim^CD16^dim^ population would accumulate for the same reasons in these patients but would, at least in part, directly stem from the original CD56^dim^ precursor. There might also be a partial block in the final maturation step of NK cells in these diseases, as previously suggested for TAP deficiency (23), leading to an increased precursor frequency in some cases.

Accordingly, the analysis of the CD56^dim^CD16^dim^ subset in HIV-infected patients highlighted that it behaves as a distinct NK subset since its distribution was, among the CD56^dim^ subpopulations, the most affected by the infection. In addition, we demonstrate that HIV-1 infection affects differentially the expression of several NK cell markers in CD56^dim^CD16^dim^ and other NK cell subsets. Based on descriptive differences of a large number of NK marker expression, we cannot ascertain that the CD56^dim^CD16^dim^ subset is either an intermediate phenotype between CD56^dim^CD16^bright^ and CD56^dim^CD16^−^ or between CD56^dim^CD16^bright^ and CD56^bright^CD16^dim^ NK cells. Nevertheless, functional data from unsorted HD and HIV-1-infected patients’ PBMC argue in favor of an intermediate state of CD56^dim^CD16^dim^ subset between CD56^dim^CD16^bright^ and CD56^dim^CD16^−^ NK cells. Importantly, the reduced percentage and impaired IFNγ production during infection indicate that CD56^dim^CD16^dim^ NK cells could account significantly for the impaired NK cell response during HIV-1 infection (53).

We found a relatively abundant CD56^dim^CD16^dim^ subset in almost all cohorts included in our study. The percentages among total NK cells often reached or even exceeded those of the CD56^bright^ populations, so that we suggest to consider the CD56^dim^CD16^dim^ population as an individualized and distinguishable subset with a characteristic phenotype.

## Ethics Statement

Blood samples were collected in accordance with the Declaration of Helsinki from the HD and the patients who each gave informed consent. The study was approved by the National Research Ethics Committee of Luxembourg (CNER, approval numbers 201109/05 and 201209/01). For the pediatric FHL II and CVID patients, parents signed the informed consent form (ethics approval numbers 07-07-111 and 10-7-311, respectively). All animal experiments were performed in accordance with the Animal Welfare Committee of LIH (protocol number LRTV 1402) and complied with the national legislation and guidelines for animal experimentation.

## Author Contributions

MA designed, performed, and analyzed experiments and drafted figures. GI and AP designed, performed, and analyzed experiments and drafted figures. MS designed, performed, and analyzed experiments. VF performed and analyzed the research with the humanized mouse model. IS performed and analyzed the research on FHL II and CVID patients. NS performed the statistical analyses. TM drafted tables and took part in the supervision and the writing of the manuscript. NA performed and analyzed the research on MM patients. BJ supervised the research on MM patients. CT-V supervised the research on FHL II and CVID patients. CS-D designed and supervised the study. JZ designed and supervised the study and wrote the manuscript. All authors participated in the preparation and editing of the manuscript.

## Conflict of Interest Statement

The authors declare that the research was conducted in the absence of any commercial or financial relationships that could be construed as a potential conflict of interest.

## Supplementary Material

Figure S1 Percentages of blood natural killer cells from the CD56^bright^CD16^dim^, CD56^dim^CD16^bright^, CD56^dim^CD16^dim^, and CD56^dim^CD16^−^ subtypes expressing the markers NKG2C, NKG2D, NKp30, SIGLEC-7, CD244, CD38, CD27, CD69, CD62L, CD8, human leukocyte antigen-DR, KLRG1, and CD226 from frozen peripheral blood mononuclear cells of a cohort of healthy donors (*n* = 12 for all markers except for KLRG1 and CD226, *n* = 6) (**p* < 0.05; ***p* < 0.01; ****p* < 0.001).

Figure S2 Dot plots from a representative healthy donor representing the percentages of blood natural killer cells from the CD56^dim^CD16^bright^, CD56^dim^CD16^dim^, and CD56^dim^CD16^−^ subtypes expressing the markers KIR2DL2/DL3/DS2, NKG2A, and CD57 from frozen peripheral blood mononuclear cells.

Figure S3 Percentages of blood natural killer cells from the CD56^bright^CD16^dim^, CD56^dim^CD16^bright^ CD56^dim^CD16^dim^, and CD56^dim^CD16^−^ subtypes expressing the markers KLRG1, CD226, and NKG2A from frozen and fresh peripheral blood mononuclear cells of a cohort of healthy donors (*n* = 5).

Figure S4 Dot plots from a representative healthy donor and an HIV-1-infected patient when viremic representing the percentages of blood natural killer cells from the CD56^dim^CD16^bright^, CD56^dim^CD16^dim^, and CD56^dim^CD16^−^ subtypes expressing the marker SIGLEC-7 from frozen peripheral blood mononuclear cells.

Figure S5 Dot plots from a representative healthy donor and an HIV-1-infected patient when viremic representing the percentages of blood natural killer cells from the CD56^dim^CD16^bright^, CD56^dim^CD16^dim^, and CD56^dim^CD16^−^ subtypes expressing the marker NKp30 from frozen peripheral blood mononuclear cells.

Figure S6 Dot plots from a representative healthy donor and an HIV-1-infected patient when viremic representing the percentages of blood natural killer cells from the CD56^dim^CD16^bright^, CD56^dim^CD16^dim^, and CD56^dim^CD16^−^ subtypes expressing the markers KIR2DL1/DS1 from frozen peripheral blood mononuclear cells.

Figure S7 **(A–D)** Flow cytometry dot plot of CD56 versus CD16 after gating on alive, single, CD3^−^CD14^−^CD19^−^ blood cells from four familial hemophagocytic lymphohistiocytosis type II (FHL II) patients, two **(A,B)** having an expanded CD56^dim^CD16^dim^ population and two others **(C,D)** not. **(E)** Percentages of the different natural killer cell subsets relative to the total NK cell population (100%) in the cohort of four FHL II patients compared to four healthy donors.

Figure S8 **(A,B)** Flow cytometry dot plot of CD56 versus CD16 after gating on alive, single, CD3^−^CD14^−^CD19^−^ blood cells from a representative healthy donor **(A)** and a representative common variable immunodeficiency (CVID) patient **(B)**. **(C)** Percentages of the different natural killer cell subsets relative to the total NK cell population (100%) in the cohort of 19 CVID patients compared to 14 healthy donors (**p* < 0.05).

Table S1 Virological and immunological characteristics of the healthy donors and the HIV-infected patients when viremic or aviremic under antiretroviral therapy.

Table S2 Antibody clones and fluorochromes used.

Table S3 Summary of the flow cytometry results.
