# Supplementary figures and images for: Human CD56dimCD16dim Cells As an Individualized Natural Killer Cell Subset
Source: Front Immunol. 2017 Jun 19;8:699. doi: 10.3389/fimmu.2017.00699 (PMC5474676; doi:10.3389/fimmu.2017.00699)

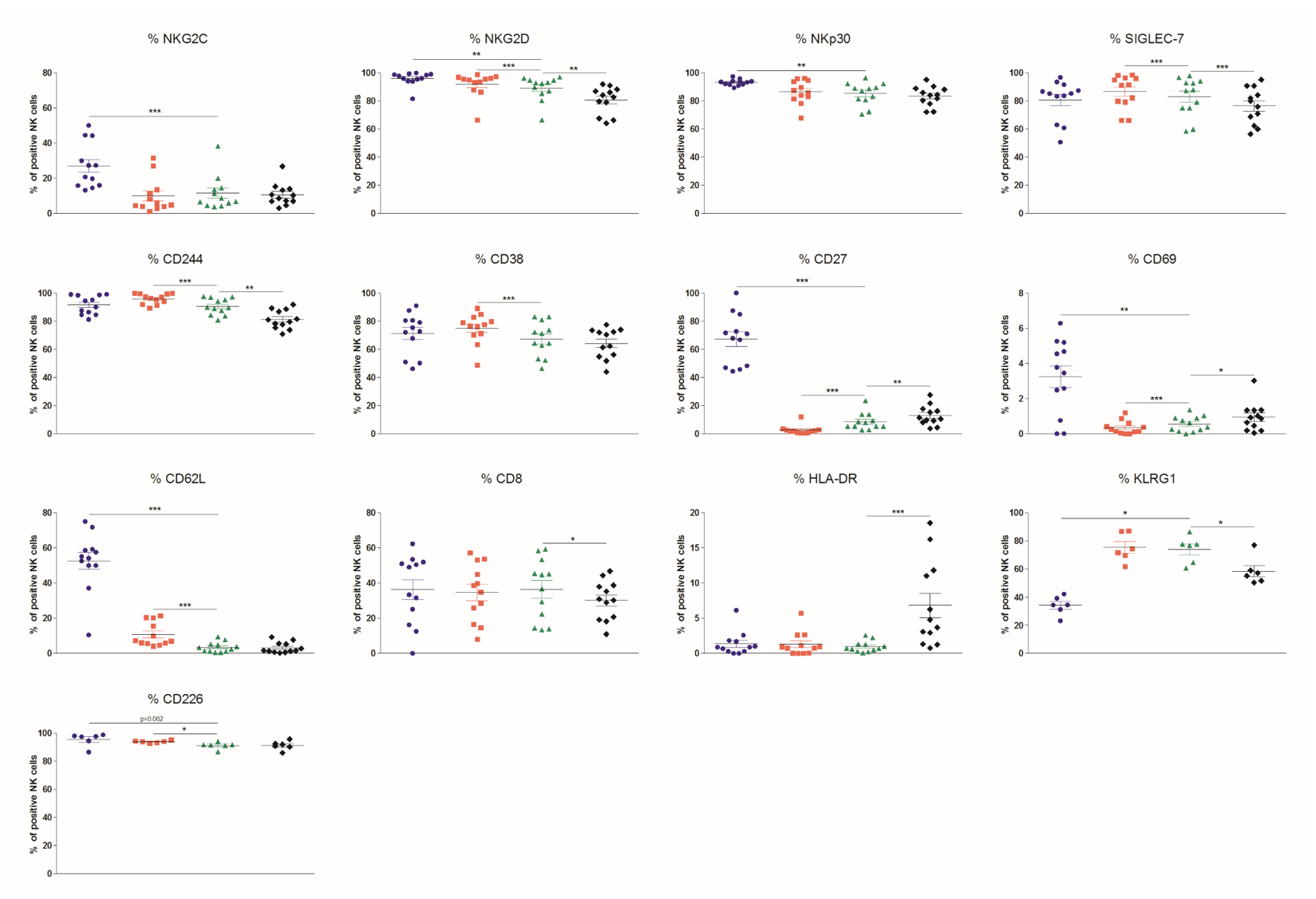

Supplement: Figure S1 — Percentages of blood natural killer cells from the CD56brightCD16dim, CD56dimCD16bright, CD56dimCD16dim, and CD56dimCD16− subtypes expressing the markers NKG2C, NKG2D, NKp30, SIGLEC-7, CD244, CD38, CD27, CD69, CD62L, CD8, human leukocyte antigen-DR, KLRG1, and CD226 from frozen peripheral blood mononuclear cells of a cohort of healthy donors (n = 12 for all markers except for KLRG1 and CD226, n = 6) (*p < 0.05; **p < 0.01; ***p < 0.001). [file Image_1.tif]

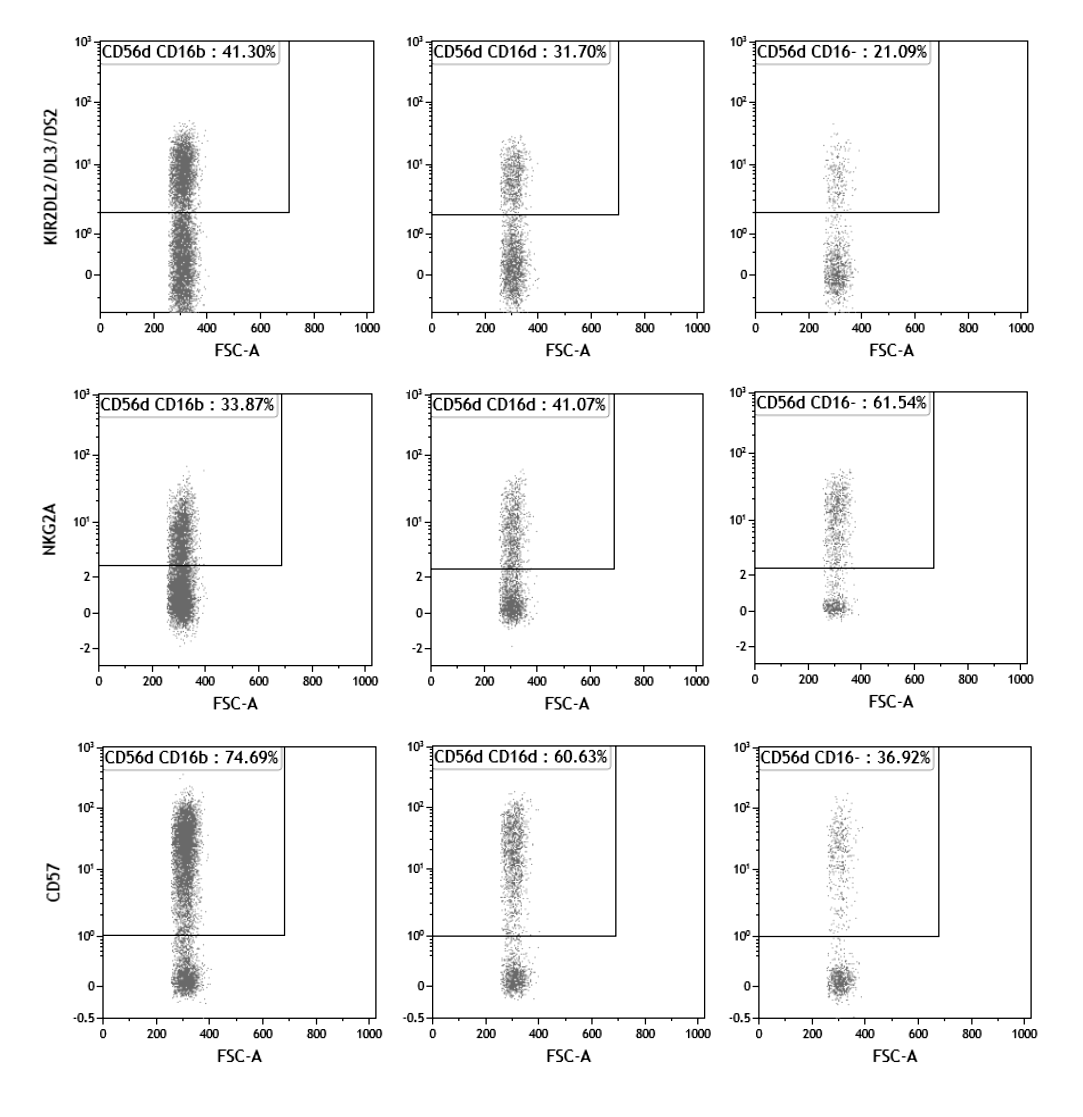

Supplement: Figure S2 — Dot plots from a representative healthy donor representing the percentages of blood natural killer cells from the CD56dimCD16bright, CD56dimCD16dim, and CD56dimCD16− subtypes expressing the markers KIR2DL2/DL3/DS2, NKG2A, and CD57 from frozen peripheral blood mononuclear cells. [file Image_2.tif]

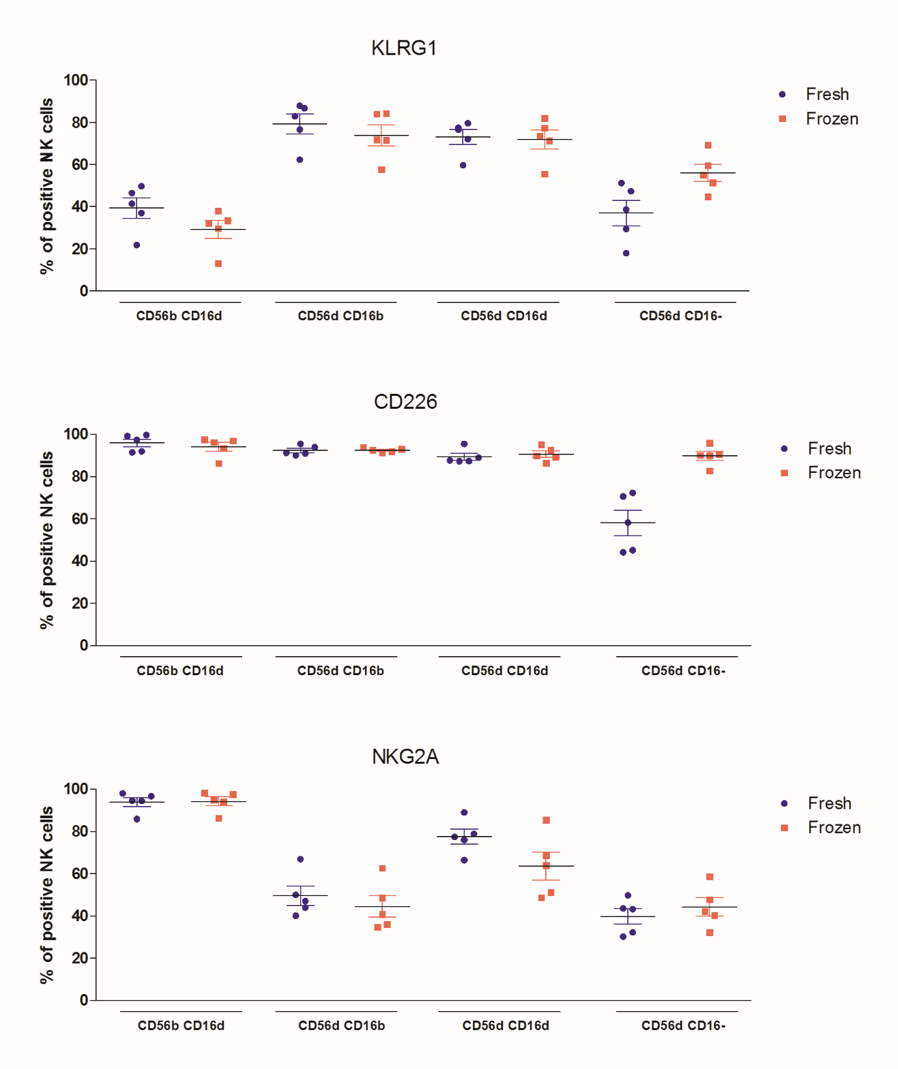

Supplement: Figure S3 — Percentages of blood natural killer cells from the CD56brightCD16dim, CD56dimCD16bright CD56dimCD16dim, and CD56dimCD16− subtypes expressing the markers KLRG1, CD226, and NKG2A from frozen and fresh peripheral blood mononuclear cells of a cohort of healthy donors (n = 5). [file Image_3.tif]

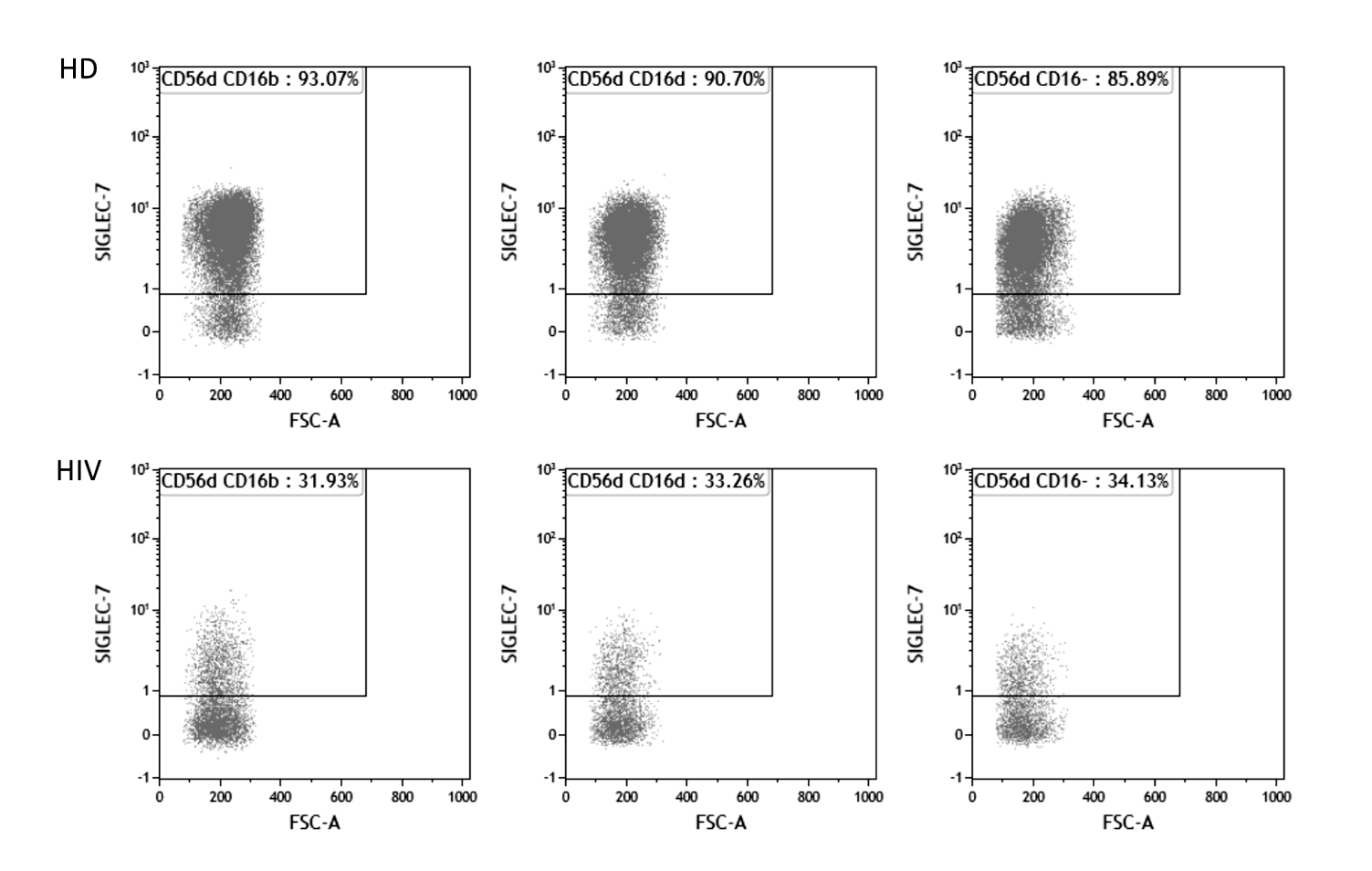

Supplement: Figure S4 — Dot plots from a representative healthy donor and an HIV-1-infected patient when viremic representing the percentages of blood natural killer cells from the CD56dimCD16bright, CD56dimCD16dim, and CD56dimCD16− subtypes expressing the marker SIGLEC-7 from frozen peripheral blood mononuclear cells. [file Image_4.tif]

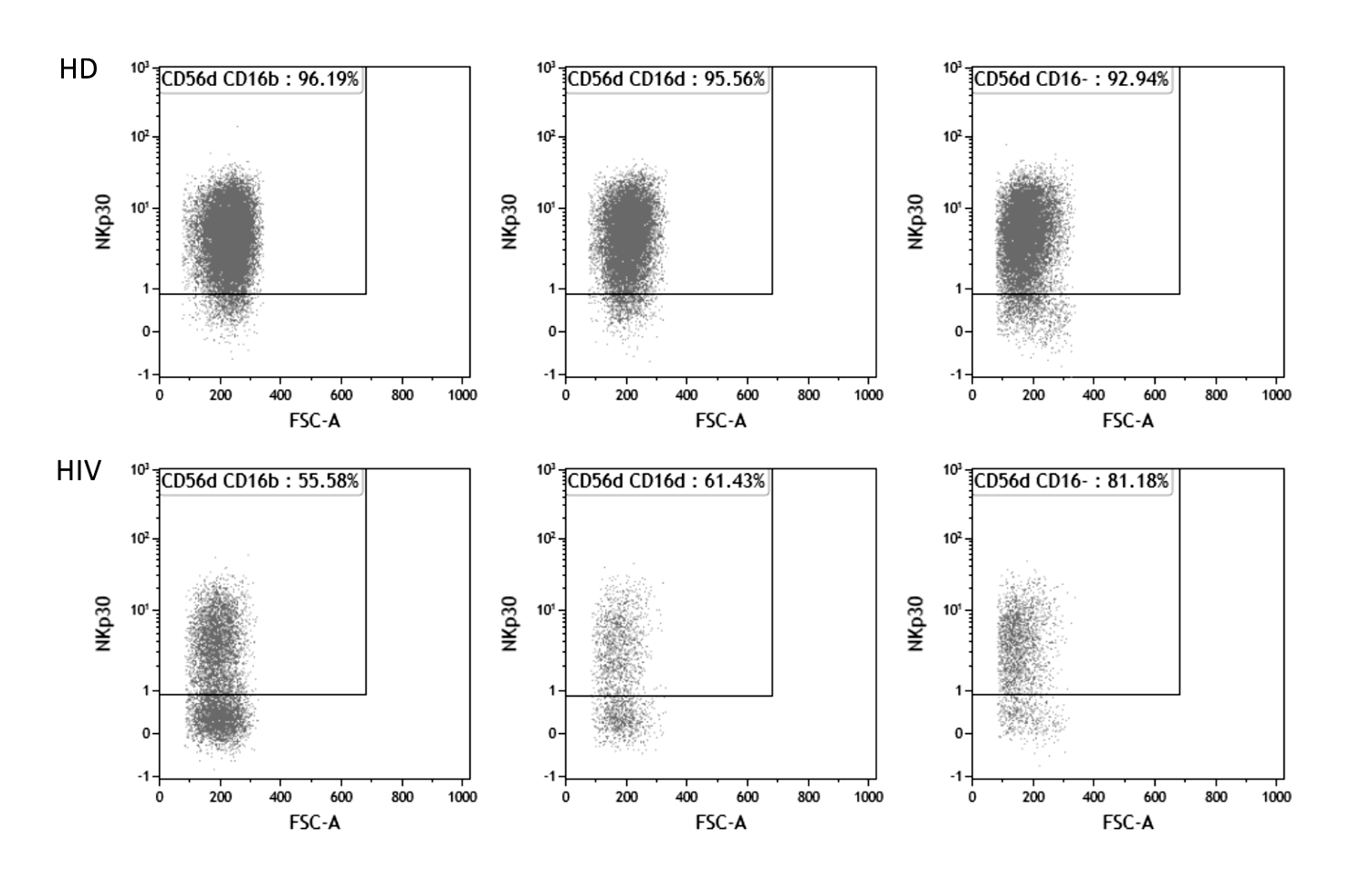

Supplement: Figure S5 — Dot plots from a representative healthy donor and an HIV-1-infected patient when viremic representing the percentages of blood natural killer cells from the CD56dimCD16bright, CD56dimCD16dim, and CD56dimCD16− subtypes expressing the marker NKp30 from frozen peripheral blood mononuclear cells. [file Image_5.tif]

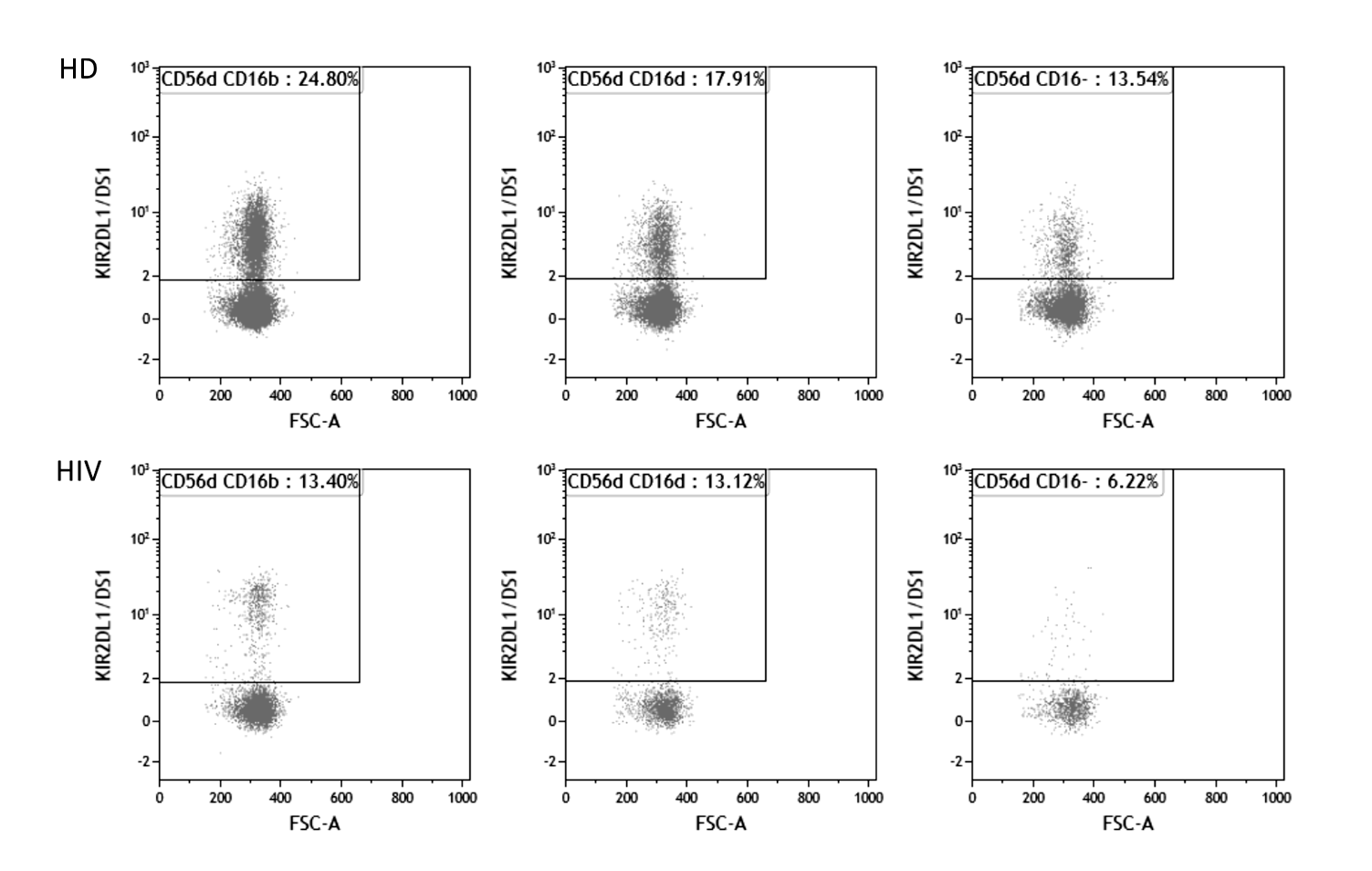

Supplement: Figure S6 — Dot plots from a representative healthy donor and an HIV-1-infected patient when viremic representing the percentages of blood natural killer cells from the CD56dimCD16bright, CD56dimCD16dim, and CD56dimCD16− subtypes expressing the markers KIR2DL1/DS1 from frozen peripheral blood mononuclear cells. [file Image_6.tif]

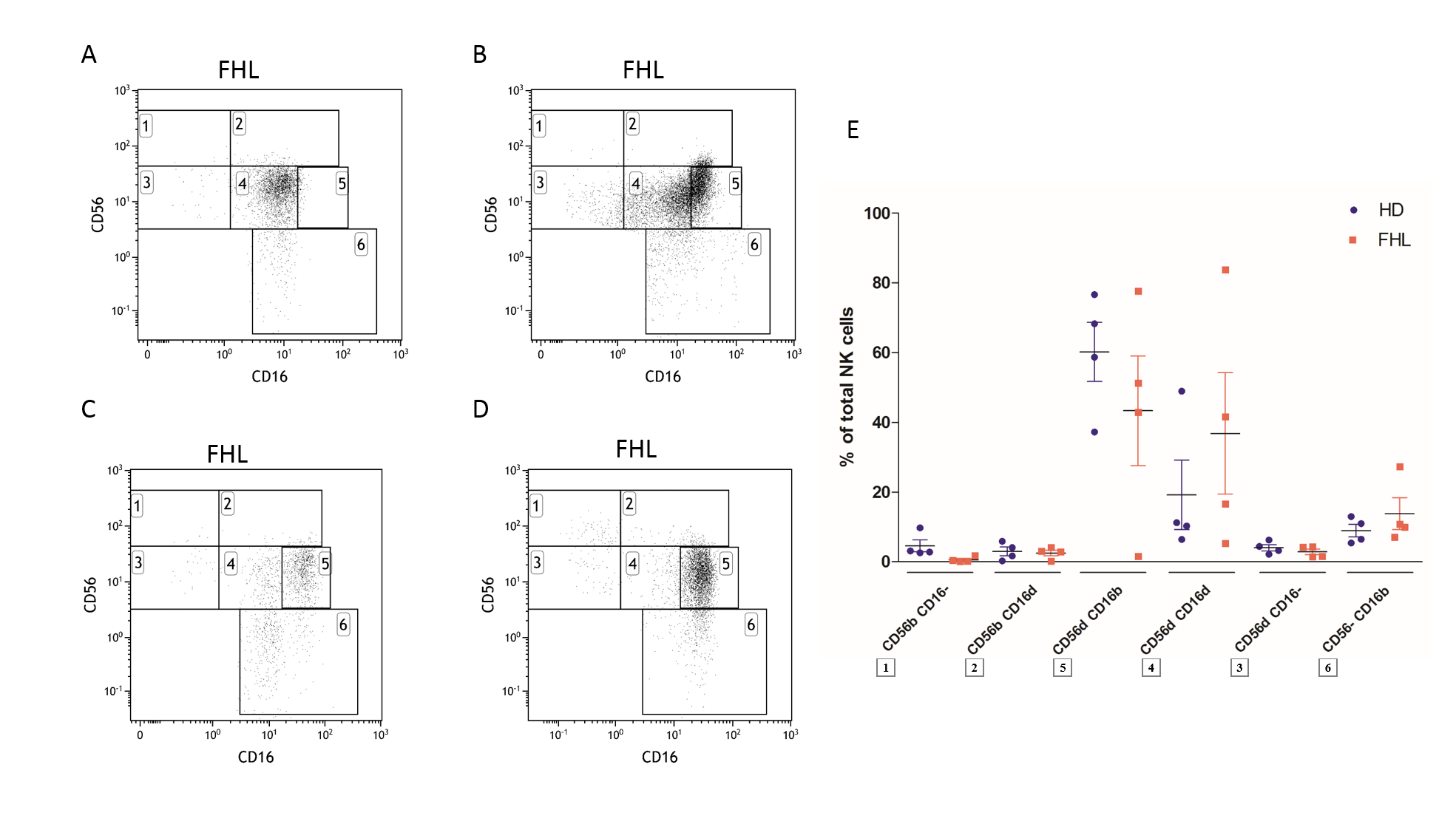

Supplement: Figure S7 — (A–D) Flow cytometry dot plot of CD56 versus CD16 after gating on alive, single, CD3−CD14−CD19− blood cells from four familial hemophagocytic lymphohistiocytosis type II (FHL II) patients, two (A,B) having an expanded CD56dimCD16dim population and two others (C,D) not. (E) Percentages of the different natural killer cell subsets relative to the total NK cell population (100%) in the cohort of four FHL II patients compared to four healthy donors. [file Image_7.tif]

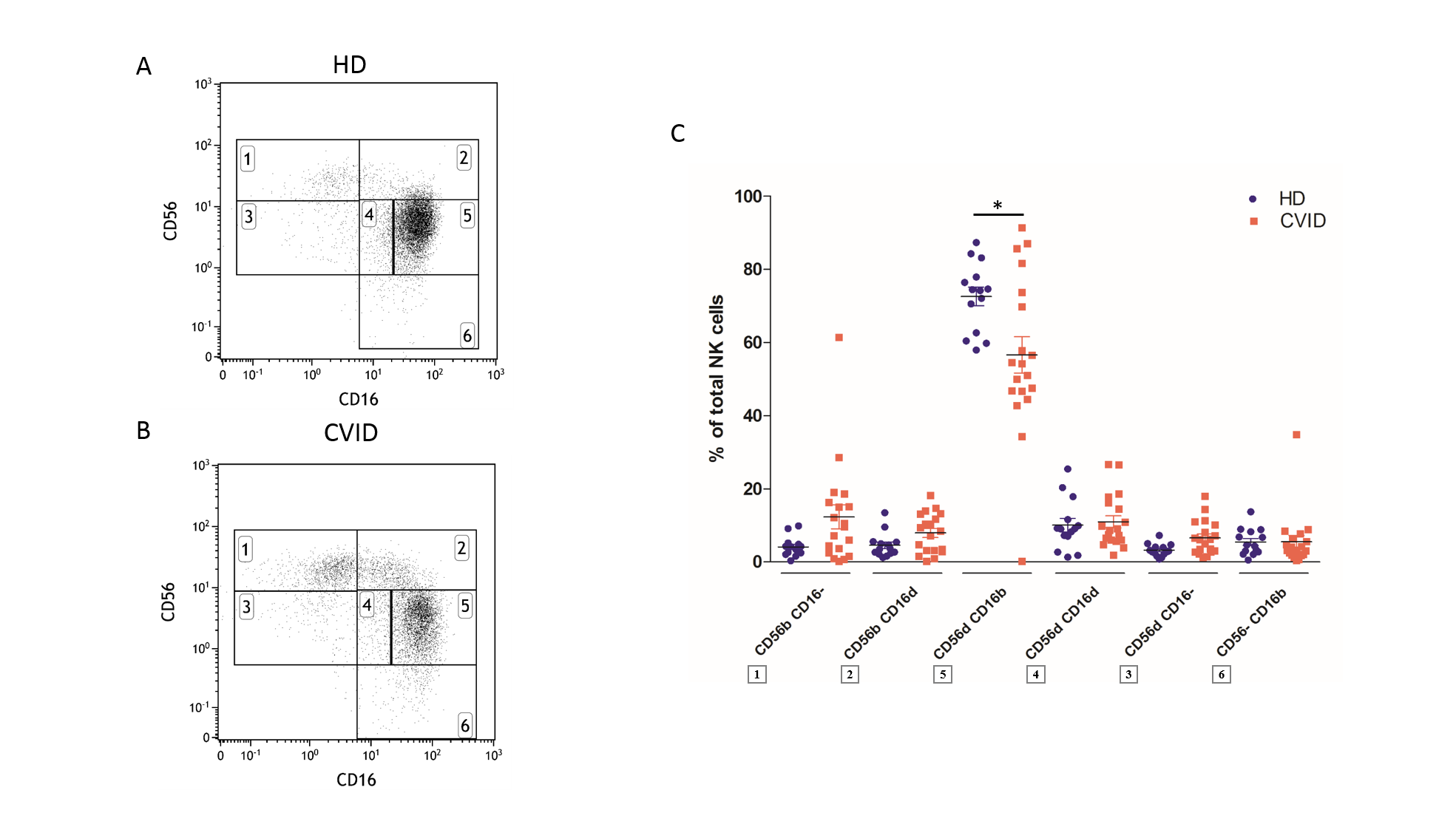

Supplement: Figure S8 — (A,B) Flow cytometry dot plot of CD56 versus CD16 after gating on alive, single, CD3−CD14−CD19− blood cells from a representative healthy donor (A) and a representative common variable immunodeficiency (CVID) patient (B). (C) Percentages of the different natural killer cell subsets relative to the total NK cell population (100%) in the cohort of 19 CVID patients compared to 14 healthy donors (*p < 0.05). [file Image_8.tif]

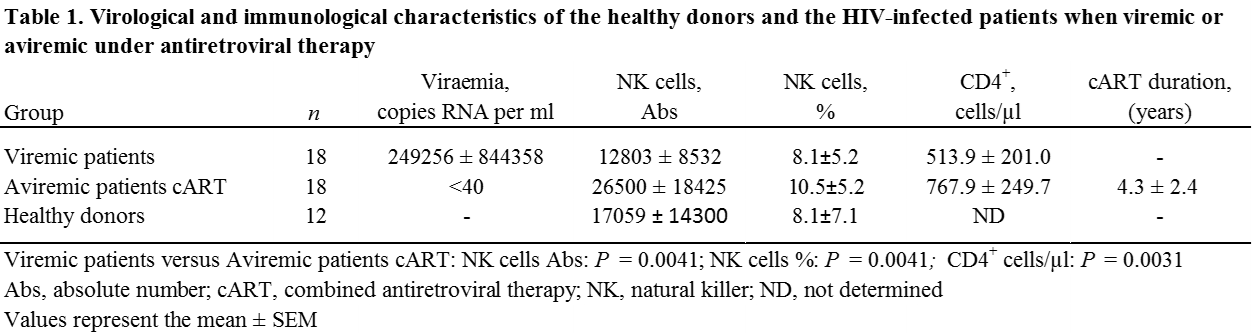

Supplement: Table S1 — Virological and immunological characteristics of the healthy donors and the HIV-infected patients when viremic or aviremic under antiretroviral therapy. [file Table_1.docx]

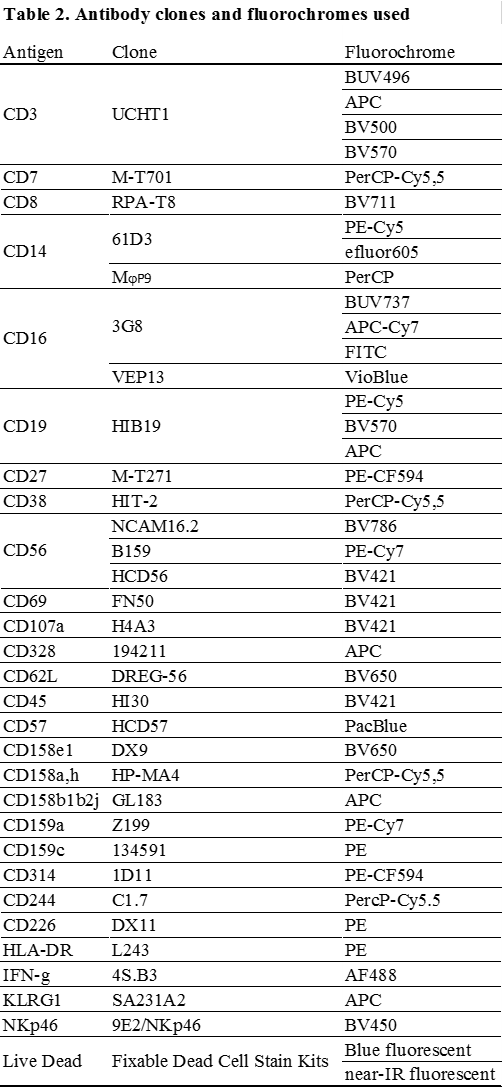

Supplement: Table S2 — Antibody clones and fluorochromes used. [file Table_2.docx]

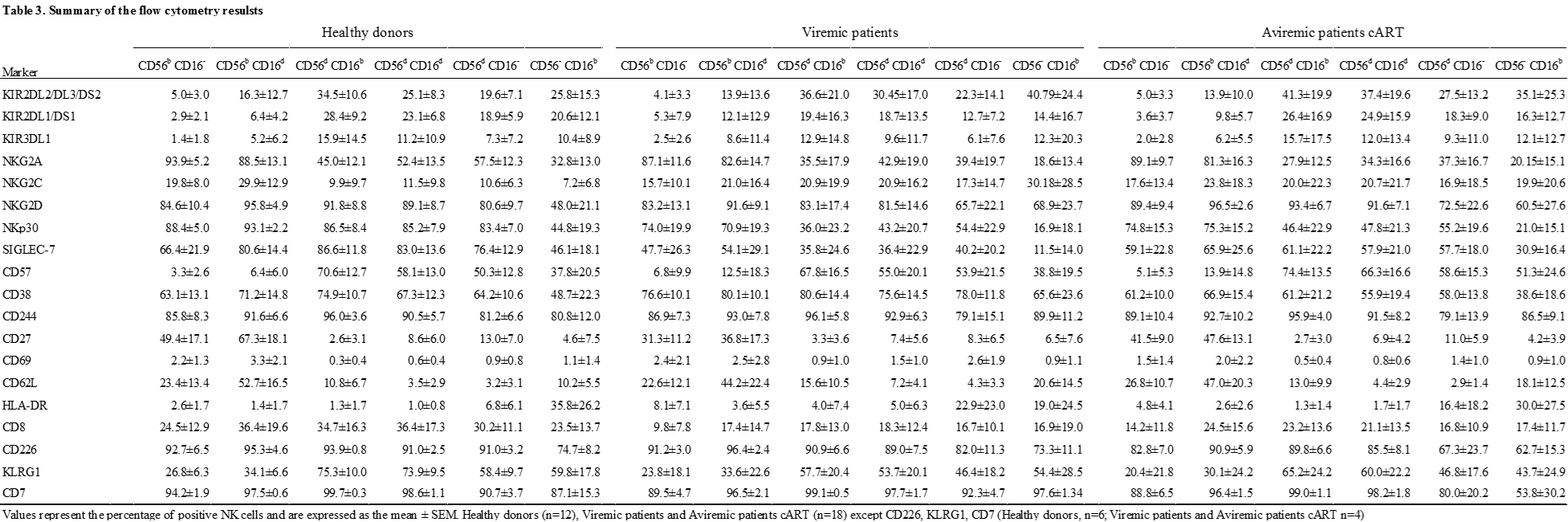

Supplement: Table S3 — Summary of the flow cytometry results. [file Table_3.docx]
